# Dual-Faced Role of GDF6 in Cancer: Mechanistic Insights into Its Context-Dependent Regulation of Metastasis and Immune Evasion Across Human Malignancies

**DOI:** 10.3390/cimb47040249

**Published:** 2025-04-02

**Authors:** Qi Zhu, Jianshu Wei, Weidong Han

**Affiliations:** 1 Department of Bio-Therapeutic, The First Medical Center, Chinese PLA General Hospital, 28 Fuxing Road, Haidian District, Beijing 100853, China; paris1028@163.com; 2 Changping Laboratory, Yard 28, Science Park Road, Changping District, Beijing 102206, China

**Keywords:** GDF6, TGF-β superfamily, epigenetic regulation, immune microenvironment, PI3K-Akt pathway

## Abstract

Growth differentiation factor 6 (GDF6), a member of the TGF-β superfamily, plays multifaceted roles in tumorigenesis, yet its molecular mechanisms and cancer-type-specific regulatory networks remain poorly defined. This study investigates GDF6’s context-dependent functions through pan-cancer multi-omics integration and functional validation. Transcriptomic data from TCGA (33 cancers, *n* = 10,535) and GTEx were analyzed to assess GDF6 dysregulation. Co-expression networks, pathway enrichment (KEGG/GO), and epigenetic interactions (m6A, m5C, m1A) were explored. Functional assays included siRNA knockdown, wound healing, and validation in immunotherapy cohorts. GDF6 exhibited bidirectional expression patterns, with downregulation in 23 cancers (e.g., GBM, BRCA) and upregulation in 7 malignancies (e.g., KIRC, PAAD). Mechanistically, GDF6 activated the PI3K-Akt/VEGF pathways, thereby promoting angiogenesis and metastasis. It modulated epigenetic regulation through interactions with m6A readers and erasers. Additionally, GDF6 reshaped the immune microenvironment by recruiting myeloid-derived suppressor cells (MDSCs) and cancer-associated fibroblasts. Notably, GDF6’s dual role extended to immunotherapy: it suppressed anti-PD1 efficacy but enhanced anti-PD-L1 sensitivity, linked to differential MHC-II and hypoxia-response regulation. This study deciphers GDF6’s context-dependent molecular networks, revealing its dual roles in metastasis and immune evasion. These findings highlight GDF6 as a central node in TGF-β-mediated oncogenic signaling and a potential therapeutic target for precision intervention.

## 1. Introduction

Despite remarkable advances in cancer immunotherapy, clinical management remains hindered by two pervasive challenges: tumor heterogeneity-driven therapeutic resistance and the lack of biomarkers to predict dichotomous responses to immune checkpoint inhibitors (ICIs). While PD-L1 expression and tumor mutational burden (TMB) guide ICI selection, up to 60% of patients exhibit innate or acquired resistance, underscoring the urgent need for biomarkers that dynamically reconcile microenvironmental plasticity with treatment outcomes [1,2].

Growth differentiation factor 6 (GDF6), a TGF-β superfamily member, emerges as a compelling candidate to address these gaps. GDF6, also known as BMP13, plays indispensable roles in embryogenesis [3], particularly in skeletal patterning [4], joint morphogenesis [5], and retinal development [6]. It orchestrates skeletal patterning, joint morphogenesis, and retinal development during embryogenesis through BMP/Smad signaling, while in tissue homeostasis, it maintains extracellular matrix integrity and suppresses inflammation by modulating pathways like p38 MAPK to counteract degeneration [7,8]. Intriguingly, these developmental mechanisms—such as SMAD-mediated transcriptional regulation and VEGF-driven angiogenesis—are frequently co-opted in malignancies [9,10,11]. For instance, aberrant GDF6 expression in tumors may hijack BMP-dependent differentiation pathways to induce cellular quiescence or amplify pro-angiogenic signals, fostering metastatic niches [3,12].

Unlike canonical TGF-β members (e.g., TGFB1) that promote angiogenesis via VEGF induction and suppress antitumor immunity through SMAD2/3 signaling, GDF6 limits angiogenesis by restraining VEGF signaling and potentially modulates immune responses through SMAD1/5-dependent mechanisms [13,14]. Preliminary evidence suggests GDF6 exerts context-dependent immunomodulatory effects. In gastric cancer, GDF6 overexpression correlates with tumor cell stemness [15], whereas in renal cell carcinoma, it paradoxically enhances VEGF-mediated vascular normalization [16]. This duality positions GDF6 at the nexus of tumor-immune crosstalk, yet its pan-cancer clinical implications remain unexplored. Critically, no existing biomarker simultaneously predicts resistance to anti-PD1 and sensitivity to anti-PD-L1—a dichotomy observed in ICI-treated patients [17]. Our pan-cancer analysis reveals GDF6’s unique capacity to stratify these opposing responses; high GDF6 expression associates with anti-PD1 refractoriness but potentiates anti-PD-L1 efficacy, likely through differential modulation of myeloid suppressive cells and antigen presentation machinery. Such bidirectional regulation could redefine patient selection paradigms, particularly in gastrointestinal and genitourinary malignancies where ICI resistance rates exceed 50% [18]. Beyond immunotherapy stratification, GDF6’s dual role in genomic instability (TMB/MSI/HRD modulation) and metastatic progression (via PI3K-Akt/VEGF pathways) further underscores its therapeutic relevance. Here, we interrogate GDF6 across 33 malignancies to address three unmet needs—prognostic stratification, therapeutic guidance, and targetability. By integrating multi-omics profiles with functional validation, this study establishes GDF6 as a biomarker with direct implications for precision immunotherapy and metastatic intervention.

## 2. Materials and Methods

### 2.1. Data Acquisition

In this study, we obtained gene expression profiles and clinical metadata from public repositories. Normal tissue expression data were sourced from the Human Protein Atlas (HPA; https://www.proteinatlas.org, accessed on 15 February 2025). Pan-cancer transcriptomics data, including 33 cancers with a total of 10,535 samples, was obtained from TCGA, and normal controls were sourced from GTEx via UCSC Xena (https://xenabrowser.net, accessed on 15 February 2025). For protein-level validation, we used TIMER2.0 (https://timer.cistrome.org, accessed on 15 February 2025) and HPA immunohistochemistry. Low-quality samples were excluded based on the following criteria: (1) samples with >50% missing expression values, (2) samples with ambiguous clinical annotations, (3) cancer types with fewer than 3 samples. Raw expression values were normalized using the DESeq2 package (v1.34.0) to correct for batch effects between TCGA and GTEx datasets. Additionally, all expression values were log2-transformed with a pseudo-count of 0.001 (log2(x + 0.001)) to stabilize variance and approximate normality for downstream analyses.

### 2.2. Expression Profiling

We performed a stage-specific analysis of GDF6 using the GEPIA2 (http://gepia.cancer-pku.cn, accessed on 15 February 2025) “Stage plot” module with log2(x + 0.001) transformed data. Differential expression was assessed by ANOVA with Tukey post hoc test using R v3.6.4, and we excluded cancers with fewer than 3 samples per stage to ensure the reliability of the results. Additionally, we explored the association between GDF6 and the immune/tumor microenvironment (TME) by applying the ESTIMATE algorithm (for stromal and immune scores) through the R package ESTIMATE (Version 2.0.0).

We analyzed the expression of GDF6 across molecular subtypes of selected cancers using the cBioPortal database (http://www.cbioportal.org/, accessed on 15 February 2025), which integrates data from large-scale cancer genomics projects, including The Cancer Genome Atlas (TCGA). GDF6 mRNA expression data (RNA-Seq V2 RSEM) were extracted for each subtype. Differences in GDF6 expression across subtypes were assessed using the Kruskal–Wallis test, followed by Dunn’s post hoc test for multiple comparisons, with significance set at *p* < 0.05. All analyses were performed within the cBioPortal platform.

We used the HPA database to investigate GDF6 protein expression in human tumor tissues. Immunohistochemical (IHC) staining intensity was quantified by calculating the percentage of cases exhibiting moderate-to-strong cytoplasmic positivity across cancer types. Staining thresholds were defined as follows; specimens with weak or negative staining were excluded, while moderate/strong cytoplasmic staining (as per HPA criteria) were classified as positive. For each malignancy, the proportion of positive cases was determined by dividing the number of moderate/strong staining samples by the total analyzed specimens, expressed as a percentage.

### 2.3. Survival Analysis

We utilized GEPIA2 to obtain the overall survival (OS) and disease-free survival (DFS) significance maps and survival plots for GDF6 across all TCGA tumors. Expression thresholds for high and low cohorts were set at the 50th percentile. The log-rank test was employed for hypothesis testing. The pan-cancer dataset, TCGA Pan-Cancer, was sourced from the UCSC database, extracting GDF6 expression data while excluding samples with zero expression or follow-up times shorter than 30 days. A log2 transformation with a pseudo-count of 0.001 was applied, and cancer types with fewer than 10 samples were removed, resulting in expression data for 39 cancer types alongside their OS data. For survival analysis, cohorts were stratified into high/low GDF6 expression and survival, we constructed groups using the median expression threshold. Univariate Cox proportional hazards regression models were constructed using the R package survival (v3.2-7) with the coxph function to assess the association between single-gene expression and prognosis. We used univariate Cox proportional hazards regression models to calculate hazard ratios (HRs) and 95% confidence intervals. Clinical covariates (e.g., tumor stage, grade) were not adjusted in this preliminary analysis, as the primary aim was to screen for tissue-specific prognostic associations of GDF6 expression. Multivariable analyses adjusting for confounders will be prioritized in future validation studies. Prognostic significance was evaluated prognostic differences using the log-rank test, with Kaplan–Meier curves generated for visualization. Right-censoring was applied to account for incomplete follow-up events. Statistical significance was defined as *p* < 0.05.

### 2.4. Immune Landscape Characterization

We used the UCSC database, MuTect2 software (Version 4.0.4.0), and R software v3.6.4 to explore the relationship between gene expression and five immune pathway markers (chemokine, receptor, MHC, immunoinhibitor, and immunostimulator). Expression data of the GDF6 gene and 60 marker genes of two types of immune checkpoint pathway genes (inhibitory and stimulatory) from the pan-cancer dataset downloaded from the UCSC database were used to calculate the Pearson correlation. We also applied the GDF6 genes and DNAss calculated from methylation profiles for each tumor to determine the tumor stemness score. The expression data of the GDF6 gene in each sample was retrieved from the pan-cancer dataset. The gene expression profile of each tumor was extracted, mapped to GeneSymbol, and then the R software package ESTIMATE was used. Stromal, immune, and ESTIMATE scores were calculated for each patient in each tumor based on gene expression, resulting in immunoinfiltration scores. The Pearson’s correlation coefficient between the gene and immunoinfiltration scores in each tumor was calculated using the corr.test function of the R package psych (version 2.1.6) to identify significant correlations. Finally, the TIMER database was employed to assess the degree of immune cell infiltration in 32 types of cancer.

### 2.5. Multi-Omics Integration

The mutational landscape of GDF6 across various cancers was analyzed using the cBioPortal (http://www.cbioportal.org/, accessed on 15 February 2025). This platform was also utilized to further investigate the type and frequency of GDF6 gene mutations in all tumors. We extracted the expression data of the GDF6 gene and 44 marker genes related to three types of RNA modifications (m1A, m5C, m6A) from each sample using the UCSC database, and performed log2 (x + 0.001) transformation on each expression value. The Pearson correlation between GDF6 and five immune pathway markers was calculated. Additionally, the MuTect2 software was applied to process samples from the TCGA level Simple Nucleotide Variation dataset. The tumor mutation burden (TMB) of each tumor was calculated using the TMB function of the R software package map tools, and the TMB and gene expression data of the samples were integrated.

### 2.6. Functional Enrichment

To investigate the interactions of GDF6, we used the STRING database (https://cn.string-db.org/, accessed on 15 February 2025) to identify experimentally determined GDF6-binding proteins in humans. The “Similar Gene Detection” module of GEPIA2 was employed to screen the top 100 GDF6-related target genes. Then, the TIMER2.0 website was utilized to obtain the expression of the top 10 target genes in each tumor and display them as heatmaps. The sangerbox3.0 database (http://sangerbox.com/home.html, accessed on 15 February 2025) was accessed to acquire the KEGG pathway and GO Biological Processes of the targeted and correlated genes. We analyzed the correlation of GDF6 with 14 cancer functional states using single-cell sequence data from the CancerSEA website (http://biocc.hrbmu.edu.cn/CancerSEA/home.jsp, accessed on 15 February 2025). Furthermore, the gene co-expression analysis of GDF6 was conducted using the LinkedOmics website (https://www.linkedomics.org/login.php, accessed on 15 February 2025), and Pearson’s test was applied to detect the correlation between GDF6 and the co-expressed genes. Due to network issues, the parsing of the above websites was unsuccessful. To access the content of these websites, please check the legality of the web page links and try again.

### 2.7. Immunotherapy Analysis

The kmplot (https://kmplot.com/analysis/, accessed on 15 February 2025) was employed to analyze the associations between the immune checkpoint blockade (ICB) treatments and GDF6. The TISMO website (http://cis.hku.hk/TISIDB/index.php, accessed on 15 February 2025) was utilized to analyze the associations between the ICB treatments or cytokine treatments and GDF6.

### 2.8. Cell Culture and Functional Assays

We performed cell culture and functional assays to explore the role of GDF6 in human trophoblast cells (htr8/svneo). Cells were cultured in RPMI-1640 medium (C11875500BT, Gibco, Grand Island, NY, USA) supplemented with 10% fetal bovine serum (FBS, 10099141, Gibco, USA) and 1% penicillin/streptomycin (15140122, Gibco, USA), incubated at 37 °C with 5% CO_2_.

For siRNA knockdown, GDF6-targeting siRNA (sense: 5′-GCUAAUACGAUCACCAUCUTT-3′; antisense: 5′-AGCUGGUGAUCGUAUUAGCTT-3′, GenePharma, Shanghai, China) and negative control siRNA (sense: 5′-UUCUCCGAACGUGUCACGUTT-3′; antisense: 5′-ACGUGACACGUUCGGAGAATT-3′, GenePharma, China) were transfected using Lipofectamine 3000 (L3000015, Invitrogen, Carlsbad, CA, USA) at a concentration of 50 nM for 48 h. Knockdown efficiency was validated by quantitative reverse transcription polymerase chain reaction (qRT-PCR) using the following primers: forward primer, 5′-CCTATCACTGCGAGGGTGTAT-3′; reverse primer, 5′-GATGGGAGTCAATTTGGTGGG-3′ (GenScript, Nanjing, China).

To assess cell migration, a wound-healing assay was conducted. Cells were seeded in 6-well plates at 2 × 10^5^ cells per well and grown to 90% confluence. A linear scratch was created using a 200 μL pipette tip, and images were captured at 0 and 6 h with an Olympus IX73 microscope. The migration area was measured using ImageJ software (v1.53) (File S1). All experiments were performed in triplicate, and data were analyzed using appropriate statistical methods.

The migration rate (%) was calculated as follows:

$$Migration\;Rate=(1-\frac{A_{t}}{A_{0}})\times 100$$

$A_{0}$: Initial wound area at 0 h.

$A_{t}$: Remaining wound area at a specific time point.

### 2.9. Prognostic and Immunotherapy Stratification Analysis

Transcriptomic and clinical data for uterine corpus endometrial carcinoma (UCEC; GSE178671), stomach adenocarcinoma (STAD; GSE236522), anti-PD1 (breast cancer, GSE194040), and anti-PD-L1 (melanoma, GSE158403) cohorts were downloaded from the Gene Expression Omnibus (GEO). Raw expression values were normalized using the R package limma (v3.54.0) were and log2-transformed. Receiver operating characteristic (ROC) curves were generated using the R package pROC (v1.18.0) to calculate the area under the curve (AUC).

## 3. Results

### 3.1. GDF6 Exhibits Aberrant Expression Across Diverse Human Malignancies

**Figure 1:**
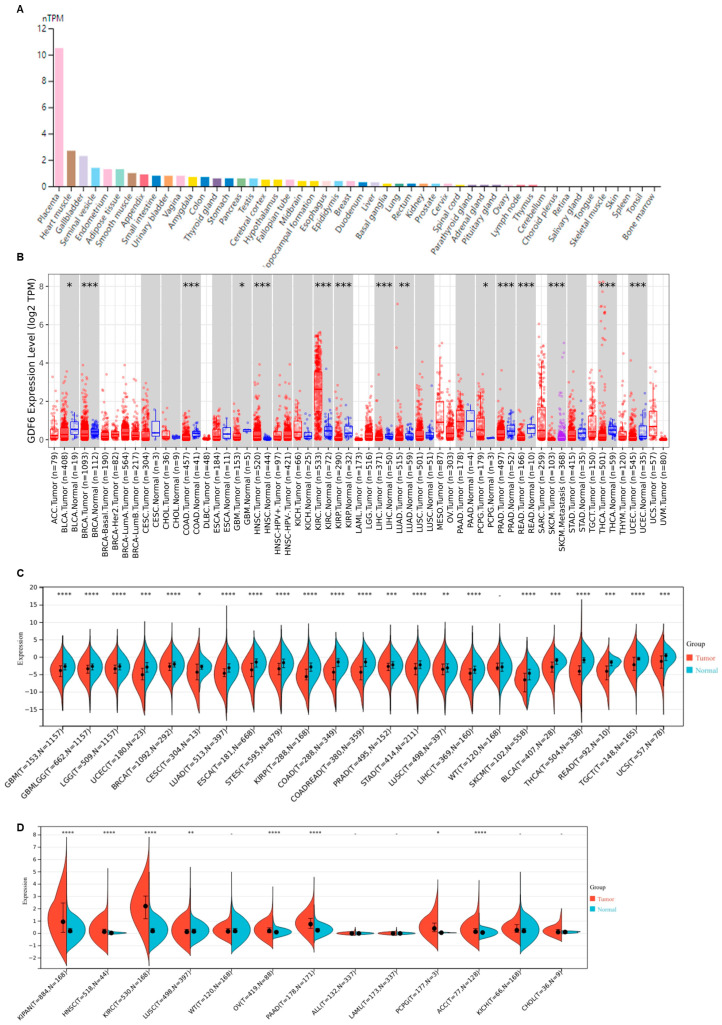
The expression of GDF6 in various human normal tissues and tumor tissues. (**A**) The mRNA expression of GDF6 in normal human tissues. (**B**) GDF6 expression in tumors and healthy tissues (TCGA database). Tumor types with significantly downregulated GDF6 expression (**C**) and those with significantly upregulated expression (**D**) in the TCGA + GTEx databases. * *p* < 0.05; ** *p* < 0.01; *** *p* < 0.001; **** *p* < 0.0001.

We first evaluated GDF6 mRNA expression across normal human tissues. GDF6 exhibited broad tissue distribution, with the highest expression observed in the placenta (Figure 1A). Moderate to high expression levels were also detected in reproductive organs (e.g., seminal vesicle, endometrium, vagina), smooth muscle-rich tissues (e.g., heart muscle, gallbladder, urinary bladder), and metabolic/immune-related organs (e.g., adipose tissue, appendix, small intestine).

Next, we integrated TCGA pan-cancer data (33 cancer types, *n* = 10,535 tumors) with GTEx normal samples to assess dysregulation of GDF6 in malignancies. Strikingly, GDF6 was significantly downregulated in 23 cancer types, including glioblastoma multiforme (GBM), brain lower grade glioma (LGG), uterine corpus endometrial carcinoma (UCEC), breast invasive carcinoma (BRCA), cervical squamous cell carcinoma and endocervical adenocarcinoma (CESC), lung adenocarcinoma (LUAD), esophageal carcinoma (ESCA), stomach and esophageal carcinoma (STES), kidney renal papillary cell carcinoma (KIRP), colon adenocarcinoma (COAD), colon adenocarcinoma/rectum adenocarcinoma esophageal carcinoma (COADREAD), prostate adenocarcinoma (PRAD), stomach adenocarcinoma (STAD), lung squamous cell carcinoma (LUSC), liver hepatocellular carcinoma (LIHC), skin cutaneous melanoma (SKCM), bladder urothelial carcinoma (BLCA), thyroid carcinoma (THCA), rectum adenocarcinoma (READ), testicular germ cell tumors (TGCT), uterine carcinosarcoma (UCS). Conversely, GDF6 showed tumor-specific upregulation in seven cancer subtypes, notably pan-kidney cohort (KIPAN), head and neck squamous cell carcinoma (HNSC), kidney renal clear cell carcinoma (KIRC), ovarian serous cystadenocarcinoma (OV), pancreatic adenocarcinoma (PAAD), pheochromocytoma and paraganglioma (PCPG), and adrenocortical carcinoma (ACC) (Figure 1B–D).

To further elucidate the role of GDF6 in cancer heterogeneity, we analyzed its expression differences across molecular subtypes, selecting BRCA, GBM, UCEC, and COADREAD as representatives due to their well-defined molecular classifications. In BRCA, significant amplifications were observed in the Basal and Her2 subtypes, whereas GBM showed frequent gains in Classical and Mesenchymal subtypes (Figure S1A,C). UCEC CN-high subtype exhibited marked amplification compared to other subtypes and COADREAD CIN subtype presented notable gains (Figure S1B,D). These subtype-specific alterations suggest that GDF6 may play an oncogenic role in tumors with genomic instability.

This bidirectional expression pattern suggests context-dependent roles of GDF6 in tumorigenesis. Its widespread suppression across carcinomas may implicate a tumor-suppressive function, while selective overexpression in specific malignancies could reflect tissue-specific oncogenic reprogramming [19]. The pronounced placental expression further hints at potential regulatory overlap between developmental pathways and cancer progression. These findings position GDF6 as a multifaceted modulator with divergent roles contingent upon tissue origin and tumor microenvironment.

To further substantiate the expression pattern of GDF6 at the protein level, immunohistochemistry (IHC) data were analyzed. As illustrated in Figure S2, GDF6 protein expression varies markedly across cancer types; melanoma exhibits the highest expression (~90% positive staining), followed by thyroid cancer with moderate expression (~50%), while prostate cancer and carcinoid show lower expression levels (~20–30%). Other cancers, such as glioma, lung cancer, colorectal cancer, lymphoma, and breast cancer, display minimal to no expression. These IHC data provide critical evidence for the bidirectional expression of GDF6 at the protein level, corroborating the mRNA expression patterns observed in this study and reinforcing its context-dependent roles in cancer.

### 3.2. GDF6 Expression Correlates with Tumor Progression and Prognosis

**Figure 2:**
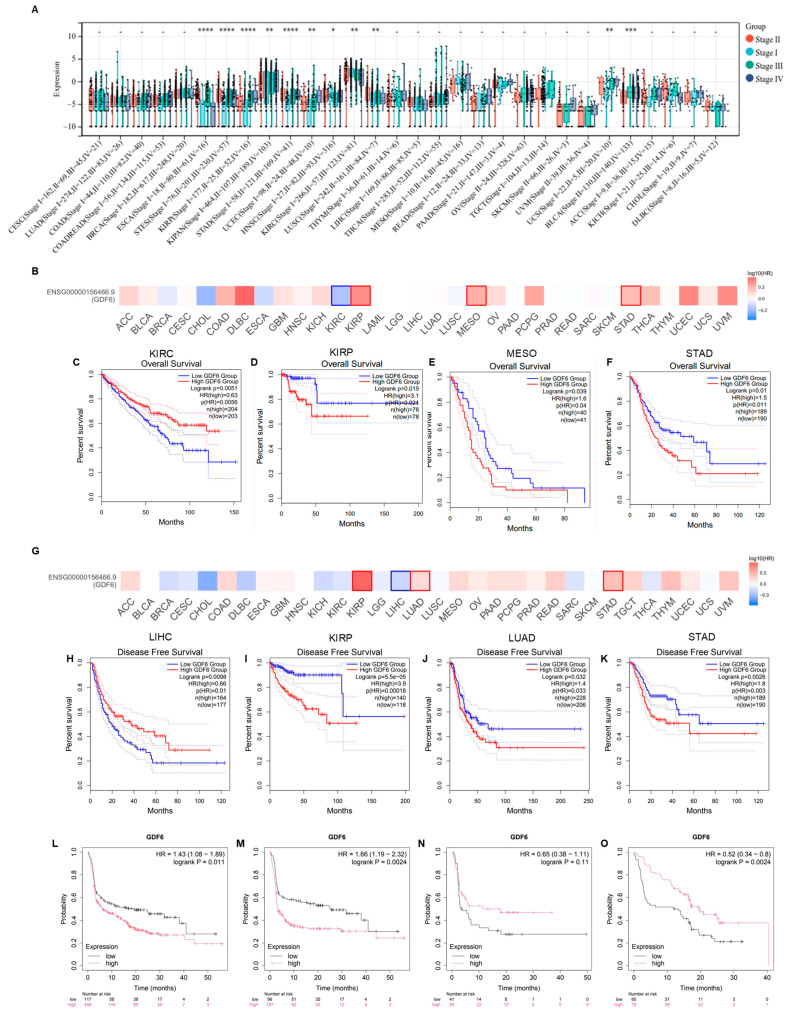
GDF6 as a multifaceted clinical biomarker: correlation with advanced staging, survival prognosis, and immunotherapy stratification. (**A**) Correlation between GDF6 expression and pathological stages of all TCGA cancers. (**B**) Relationship between GDF6 gene expression and overall survival. (**C**–**F**) Kaplan–Meier curves showing significant survival analysis results of (**B**). (**G**) Relationship between GDF6 gene expression and disease-free survival. (**H**–**K**) Kaplan–Meier curves showing significant survival analysis results of (**G**). (**L**) PFS of patients without any immunotherapy. PFS of patients treated with all anti-PD1 (**M**), anti-CTLA-4 (**N**), and anti-PD-L1 (**O**). * *p* < 0.05; ** *p* < 0.01; *** *p* < 0.001; **** *p* < 0.0001.

To systematically evaluate the role of GDF6 in cancer progression, we first integrated TCGA pan-cancer data (33 cancers, *n* = 10,535) to analyze the correlation between its expression and pathological stage. The results showed that in 11 types of tumors, GDF6 expression exhibited significant stage-dependent differences (Figure 2A), and the regulatory direction was cancer-specific. GDF6 expression increased with pathological grade in KIRP, BRCA, UCEC, and STAD, while it decreased with higher tumor grade in KIRC (Supplementary Figure S3). Survival analysis using univariate Cox regression revealed tissue-specific prognostic roles of GDF6 (Figure 2B). High GDF6 expression predicted shorter overall survival (OS) in KIRP, MESO, and STAD (Figure 2D–F). However, in KIRC, high GDF6 expression was associated with prolonged OS (Figure 2C). Similarly, GDF6 expression correlated with disease-free survival (DFS) (Figure 2G). High GDF6 predicted reduced DFS in KIRP, LUAD, and STAD (Figure 2I–K). In LIHC, high GDF6 expression improved DFS (Figure 2H). Multivariate analysis across four survival metrics (OS, DSS, DFI, PFI) identified GDF6 as a pan-prognostic risk factor in gastrointestinal (ESCA, STES, COAD/COADREAD, STAD) and genitourinary malignancies (KIRP, UCEC), with KIRP exhibiting the most consistent associations (Supplementary Figure S4A–D). Mechanistically, GDF6’s dual association with advanced staging and poor survival in KIRP and STAD suggests its role in promoting metastatic progression, potentially through extracellular matrix remodeling [16]. These findings nominate GDF6 as a novel therapeutic target for tumors with high metastatic potential, particularly via inhibition of TGF-β/SMAD signaling [15].

In the TCGA immunotherapy cohort, GDF6 exhibited divergent roles in ICI response (Figure 2L–O). High GDF6 expression correlated with shorter progression-free survival (PFS) in anti-PD1-treated patients (Figure 2M). Conversely, high GDF6 predicted improved PFS in anti-PD-L1-treated cohorts (Figure 2O). Validation in the EMT6 tumor model (TISMO database) confirmed that GDF6 expression increased in responders following anti-PD-L1 treatment (Supplementary Figure S5). Furthermore, GDF6 remained predictive in combination therapy (anti-TGFβ + anti-PD-L1), supporting its potential as an independent biomarker for immunotherapy stratification. These findings indicate the dual role of GDF6 by resistance in anti-PD1 therapy and sensitivity in anti-PD-L1 therapy. GDF6 has demonstrated its potential as an immunotherapy response biomarker for stratifying immunotherapy patients, such as prioritizing those with high expression for anti-PD-L1 therapy.

### 3.3. GDF6 Regulates Tumor Microenvironment and Stemness Features in Pan-Cancer Analysis

**Figure 3:**
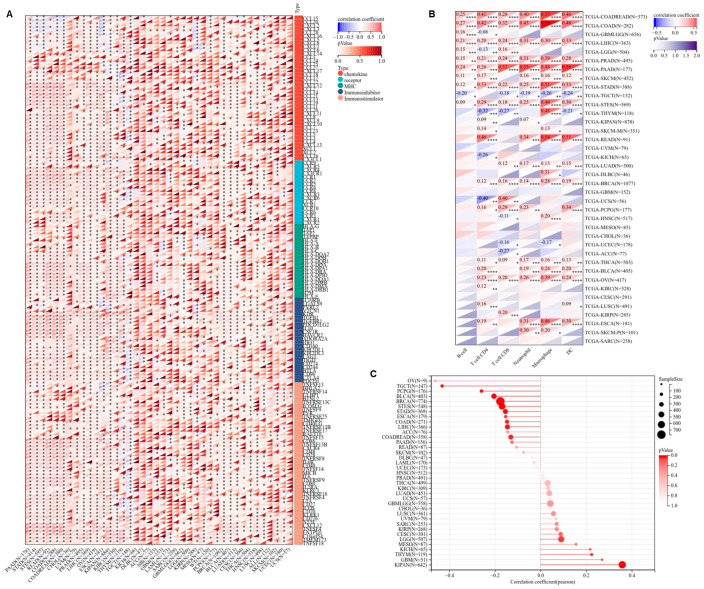
Correlation analysis of GDF6 with immune regulatory genes, immune cell infiltration, and tumor stemness score. (**A**) Correlation between GDF6 expression and the majority of immune regulatory genes. (**B**) Correlation between GDF6 and the infiltration levels of six types of immune cells, including B cells, T cells (CD4+ and CD8+), neutrophils, macrophages, and dendritic cells (DCs). (**C**) Correlation analysis between GDF6 expression and tumor stemness score. * *p* < 0.05; ** *p* < 0.01; *** *p* < 0.001; **** *p* < 0.0001.

Firstly, we investigated the correlation between GDF6 and immune-regulatory genes. Based on the UCSC pan-cancer dataset, Pearson correlation analysis revealed that GDF6 expression was positively correlated with marker genes of five immune pathways (chemokines, receptors, MHC, immune inhibitors, and immune stimulators) (Figure 3A). Subsequently, we assessed the immune infiltration levels in 38 types of tumors and found that GDF6 was significantly associated with the abundance of immune cells, including B cells, T cells (CD4+ and CD8+), neutrophils, macrophages, and dendritic cells (Figure 3B). Furthermore, based on the tumor stemness index evaluated by methylation characteristics, GDF6 was significantly associated with stemness features in 13 types of tumors. Among these, GDF6 was positively correlated in 3 tumor types, such as LGG, THYM, and KIPAN, and negatively correlated in 10 tumor types, including COAD, COADREAD, BRCA, ESCA, STES, STAD, LIHC, TGCT, PCPG, and BLCA (Figure 3C).

**Figure 4:**
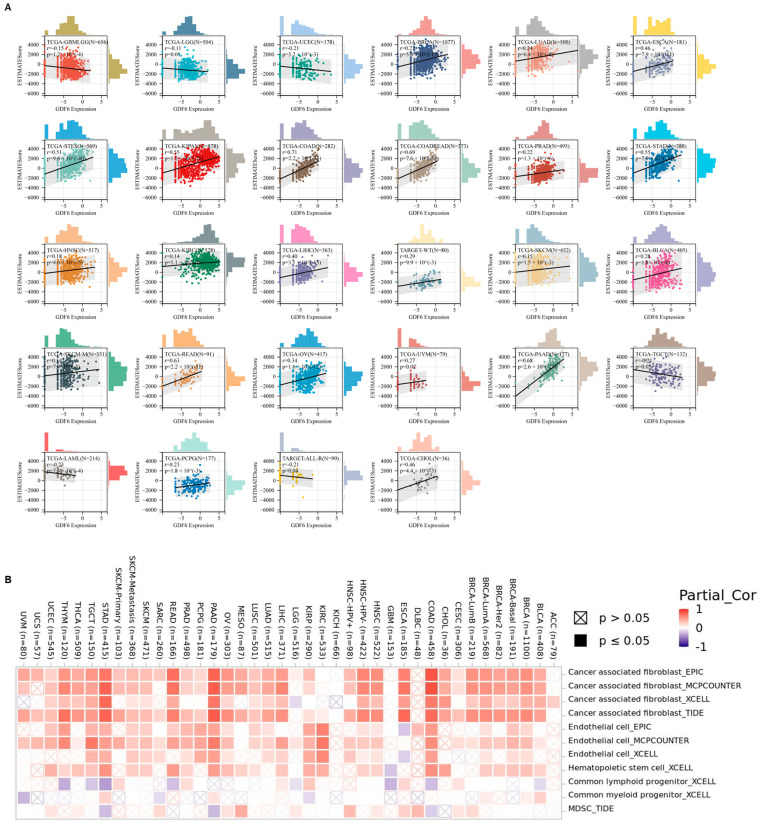
Immune infiltration analysis. (**A**) Analysis of GDF6 expression and immune infiltration scores in tumor type. (**B**) Correlation between GDF6 expression and immune infiltration levels in all TCGA cancers using the TIMER2 algorithm.

Subsequently, we observed the correlation between GDF6 and tumor immune infiltration. We found that GDF6 expression was significantly correlated with immune infiltration in 28 types of cancer, with 22 showing a positive correlation, such as BRCA, LUAD, ESCA, STES, KIPAN, COAD, COADREAD, PRAD, STAD, HNSC, KIRC, LIHC, TARGET-WT, SKCM, BLCA, SKCM-M, READ, OV, UVM, PAAD, PCPG, and CHOL, and 6 showing a negative correlation, including GBMLGG, LGG, UCEC, TGCT, LAML, and TARGET -ALL-R (Figure 4A). Further analysis revealed that in tumors with high GDF6 expression, the infiltration of the following cells was significantly increased: myeloid derived suppressor cells, hematopoietic stem cells, cancer associated fibroblasts, and endothelial cells (Figure 4B).

This suggests that GDF6 is specifically related to the regulation of immune cell subpopulations. The above studies show that the immune regulation of GDF6 exhibits significant cancer-type heterogeneity.

### 3.4. GDF6 Genomic Characteristics

**Figure 5:**
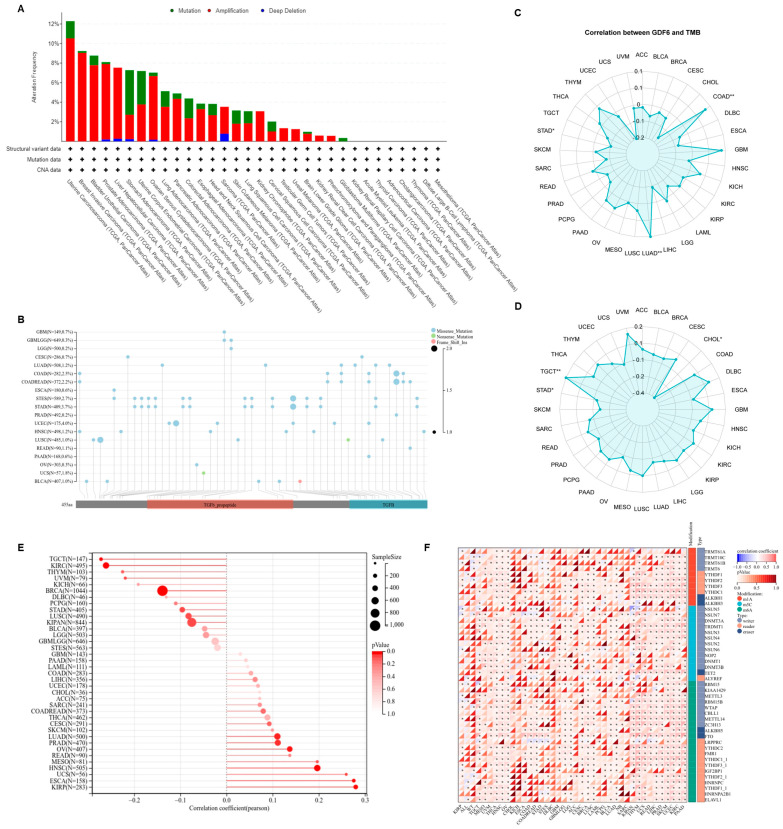
Analysis of the relationship between GDF6 expression and cancer genome instability. (**A**) Analysis of GDF6 gene alteration features (mutations, amplifications, and deep deletions) in 32 different tumors from the TCGA database. (**B**) Pan-cancer GDF6 SNV landscape, including missense mutations, frameshift insertions, and splice site mutations. Pan-cancer analysis of the correlation between GDF6 and TMB (tumor mutational burden) (**C**) and MSI (microsatellite instability) (**D**). (**E**) Analysis of the correlation between genomic HRD (homologous recombination deficiency) and GDF6 expression. (**F**) Correlation analysis of the expression of GDF6 and 44 class III RNA modification genes (m1A (10), m5C (13), m6A (21)) in each sample. * *p* < 0.05; ** *p* < 0.01.

Based on the mutation spectrum analysis of GDF6 in the TCGA pan-cancer dataset, GDF6 was found to have somatic mutations in 32 types of cancer, with the highest mutation frequency in uterine carcinosarcoma (UCS) at 12.28% (Figure 5A). The mutation types were predominantly missense mutations, mainly enriched in the TGF-β domain, potentially interfering with ligand-receptor binding (Figure 5B). To explore the impact of GDF6 on tumor genomic stability, we assessed its correlation with TMB, MSI, and HRD. High expression of GDF6 was significantly positively correlated with increased TMB in COAD and LUAD, suggesting that it may increase mutation accumulation by inducing replication stress (Figure 5C). GDF6 expression was positively correlated with MSI in TGCT, possibly reflecting its regulation of the mismatch repair pathway (Figure 5D). GDF6 was significantly positively correlated with HRD in six types of cancer, such as LUAD, ESCA, KIRP, PRAD, HNSC, and OV, and significantly negatively correlated in three types of cancer, such as BRCA, KIRC, and TGCT, suggesting that it may affect homologous recombination repair efficiency through tissue-specific mechanisms (Figure 5E). Analysis of GDF6 in relation to three RNA modification-associated genes (m1A, m5C, and m6A) revealed a significant positive correlation with the expression of m6A regulators (Figure 5F), suggesting that the epigenetic regulatory network may drive tumor progression by affecting GDF6 function.

### 3.5. Co-Expression Network, Functional Regulatory Mechanisms, and Therapeutic Sensitivity of GDF6

**Figure 6:**
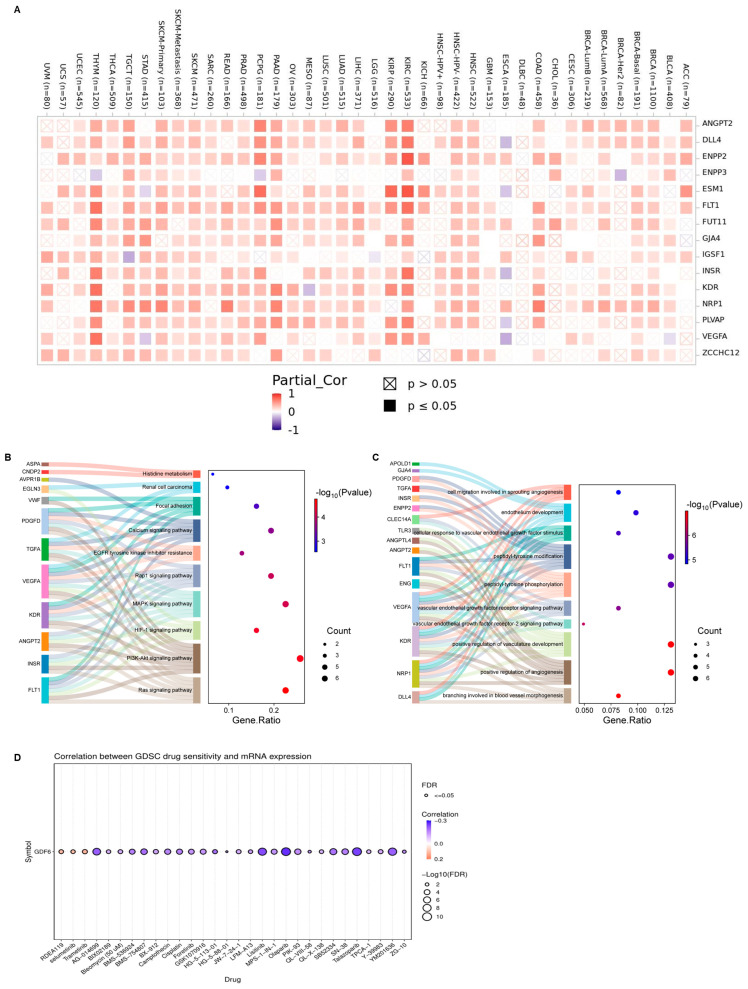
GDF6-related gene enrichment analysis and drug sensitivity analysis. (**A**) Expression of the top 13 GDF6-related target genes in cancers. (**B**) KEGG pathway analysis based on GDF6 interactions and related genes. (**C**) GO analysis based on GDF6 interactions and related genes. (**D**) Correlation between GDF6 mRNA expression and drug sensitivity in cancer cell lines (GDSC database).

To further explore the mechanisms underlying the role of GDF6 in cancer development, we used GEPIA2 to identify the top 100 genes that are significantly co-expressed with GDF6 (Supplementary Table S1). The top 13 GDF6-related target genes (such as VEGFA, FLT1, and KDR) showed positive correlations with the tissue expression in various cancer types (Figure 6A), suggesting a conserved regulatory network. Pathway enrichment analysis revealed that GDF6-associated genes are significantly enriched in oncogenic signaling pathways, including KEGG pathways like PI3K-Akt signaling, HIF-1 signaling, MAPK signaling, and EGFR tyrosine kinase inhibitor resistance (Figure 6B) and GO biological processes like angiogenesis (e.g., blood vessel morphogenesis, VEGF receptor signaling), endothelial cell development, and cell migration (Figure 6C). These findings suggest that GDF6 promotes tumor progression through synergistic mechanisms. GDF6 upregulates the VEGFR/PI3K-Akt pathway, enhancing endothelial cell recruitment and vascularization [20], and collaborates with HIF-1α to regulate hypoxia responses and recruit cancer-associated fibroblasts (CAFs), fostering an immunosuppressive niche [21].

To explore GDF6’s therapeutic implications, we analyzed its correlation with drug sensitivity using the GDSC database. GDF6 expression exhibited significant negative correlations with sensitivity to PI3K inhibitors (PIK-93; Figure 6D), indicating that tumors with elevated GDF6 levels are more resistant to PI3K-targeted therapies. This aligns with our mechanistic findings that GDF6 amplifies PI3K-Akt signaling, providing a plausible explanation for its role in driving therapeutic resistance. These results nominate GDF6 as a predictive biomarker for PI3K inhibitor resistance, with direct implications for patient stratification.

### 3.6. In Vitro Functional Validation and Clinical Biomarker Potential of GDF6

**Figure 7:**
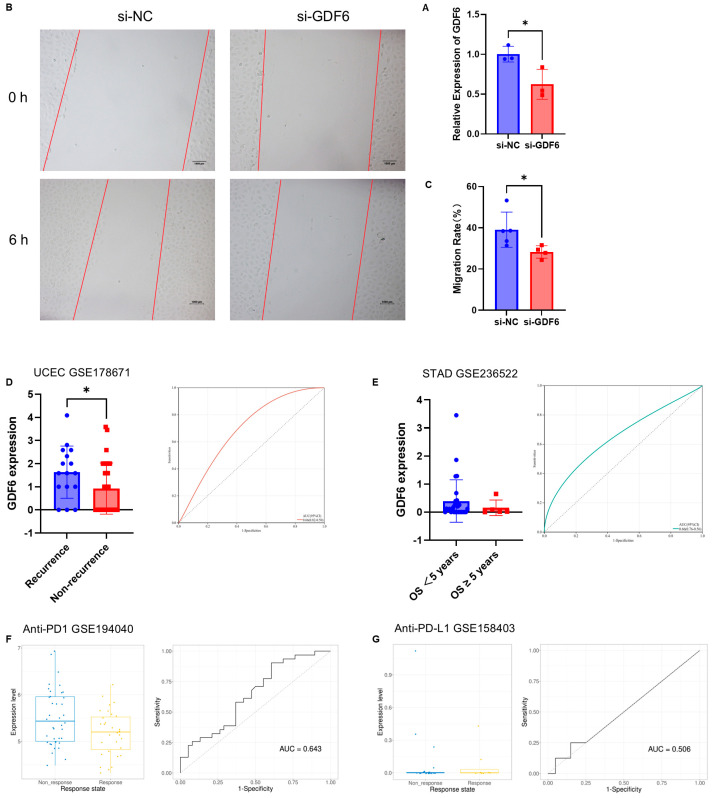
Functional validation and clinical biomarker potential of GDF6 (**A**) GDF6 knockdown in Htr8/svneo cells was confirmed by qRT-PCR. (*n* = 3). (**B**,**C**) Wound-healing assay demonstrated that GDF6 silencing significantly inhibited cell migration (si-NC group, *n* = 5; si-GDF6 group, *n* = 4, scale bar = 1000 μm). (**D**) GDF6 expression was higher in recurrent versus non-recurrent UCEC patients, with ROC analysis showing predictive accuracy (recurrent group, *n* = 24; non-recurrent group, *n* = 25, AUC = 0.66). (**E**) In STAD, GDF6 levels were elevated in patients with OS < 5 years (OS < 5 years group, *n* = 28; OS ≥ 5 years group, *n* = 5, AUC = 0.66). (**F**,**G**) GDF6 expression stratified immunotherapy response: high GDF6 correlated with anti-PD1 resistance (non-response group, *n* = 38; response group, *n* = 31, AUC = 0.643) but enhanced anti-PD-L1 sensitivity (non-response group, *n* = 20; response group, *n* = 8, AUC = 0.506). ROC curves and boxplots (median ± IQR) are shown for predictive performance. Mean ± SD; Unpaired t test; * *p* < 0.05.

To verify the oncogenic function of GDF6, we knocked down GDF6 expression in human trophoblast cells (htr8/svneo) using siRNA (Figure 7A). The scratch assay revealed that silencing GDF6 inhibited cell migration (Figure 7B,C), supporting its role in promoting tumor invasion.

Regarding the prognostic predictive value of GDF6, in the UCEC cohort (GSE178671), GDF6 expression was significantly higher in patients with recurrence compared to non-recurrent cases (AUC = 0.66, Figure 7D). In the STAD cohort (GSE236522), elevated GDF6 levels were associated with poor survival (OS < 5 years; AUC = 0.66, Figure 7E).

To assess GDF6’s role in immunotherapy stratification, we analyzed two independent cohorts. In the Anti-PD1 cohort, derived from breast cancer patients treated with Paclitaxel + Pembrolizumab, high GDF6 expression was found to be associated with treatment resistance (AUC = 0.643, Figure 7F). High GDF6 expression predicted improved response in anti-PD-L1 Cohort, derived from melanoma patients treated with Durvalumab + Trametinib (AUC = 0.506, Figure 7G).

These results indicate that GDF6 directly drives tumor invasion by promoting cell migration. Combined with the previous ICI analysis, targeting GDF6 may inhibit metastasis and improve immunotherapy response. These findings confirm GDF6’s dual role in immunotherapy: it serves as a negative predictor for anti-PD1 efficacy but a positive biomarker for anti-PD-L1 response, highlighting its potential for patient stratification in combination therapies.

## 4. Discussion

Single-gene pan-cancer analysis integrates molecular features across cancer types, revealing the conserved mechanisms and tissue-specific regulatory patterns of genes in tumors. This approach overcomes the limitations of studying a single cancer type, providing theoretical basis for the discovery of universal therapeutic targets, understanding functional heterogeneity, and guiding precision medicine [22].

Growth and differentiation factor 6 (GDF6), a member of the TGF-β superfamily, regulates skeletal formation and cell differentiation during embryonic development [23]. However, its pleiotropic effects in tumors have not been fully elucidated. In this study, we systematically explored the complex roles of GDF6 in tumor progression, immune microenvironment regulation, and treatment response by integrating pan-cancer multi-omics data and functional experiments. Our findings reveal the dual value of GDF6 as a prognostic biomarker and a potential therapeutic target.

In this study, we found that the expression pattern of GDF6 is significantly cancer-type-dependent. GDF6 was significantly downregulated in 23 types of tumors, including glioblastoma multiforme (GBM) and breast invasive carcinoma (BRCA), while it was abnormally upregulated in seven types of tumors, such as kidney renal clear cell carcinoma (KIRC) and pancreatic adenocarcinoma (PAAD). This heterogeneity may result from the dual regulation of epigenetics and genomic variations. In colorectal cancer, the high methylation of the GDF6 promoter leads to its transcriptional silence, suggesting that epigenetic silencing may mediate the inactivation of tumor-suppressor functions [24,25]. In ovarian cancer (OV), the amplification of chromosome 8q22.1 drives the overexpression of GDF6, which may promote metastasis by activating the BMP/Smad pathway [26]. GDF6 exhibits significant expression differences across cancer molecular subtypes, particularly pronounced in subtypes characterized by genomic instability. In breast cancer (BRCA), the Basal and Her2 subtypes show marked GDF6 upregulation, while in glioma (GBM), the Classical and Mesenchymal subtypes display frequent gains. Similarly, the CN-high subtype of uterine corpus endometrial carcinoma (UCEC) and the chromosomal instability (CIN) subtype of colon adenocarcinoma/rectum adenocarcinoma (COADREAD) present notable GDF6 amplification. These subtypes are often associated with aggressive behavior and poor prognosis, suggesting that GDF6 may promote angiogenesis and tumor progression by activating the PI3K-Akt and VEGF pathways [13,27]. Further clinical correlation analysis indicated that high GDF6 expression was significantly associated with advanced pathological stages and poor prognosis in endometrial cancer (UCEC) and gastric adenocarcinoma (STAD). Especially in renal papillary cell carcinoma (KIRP), the overall survival period of patients with high GDF6 expression was significantly shortened. This cross-cancer association suggests that GDF6 can serve as a biomarker for dynamically monitoring tumor progression. However, its functional direction (oncogenic or tumor-suppressive) is highly dependent on the tissue microenvironment context. Notably, our survival analyses were based on univariate Cox models without adjustment for clinical covariates (e.g., tumor stage or treatment history), which limits causal interpretation of GDF6’s prognostic role. However, similar unadjusted approaches have been widely adopted in exploratory pan-cancer studies to prioritize candidate biomarkers for further validation [28]. Future studies integrating multivariable-adjusted models and experimental validation are warranted to confirm GDF6 as an independent prognostic factor.

The immunosuppressive properties of the tumor microenvironment (TME) are key factors in immune treatment resistance. In this study, we found that GDF6 plays a multifaceted role in tumor progression through cancer-specific immune regulation, stemness remodeling, and microenvironment interaction networks. Its expression heterogeneity and functional plasticity provide new targets for precision medicine. In solid tumors such as mesothelioma (MESO) and esophageal carcinoma (ESCA), high expression of GDF6 was significantly positively correlated with the infiltration of myeloid derived suppressor cells (MDSCs) and cancer associated fibroblasts (CAFs), showing pro-immunosuppressive effects. Mechanistically, GDF6 may upregulate CXCL12 to recruit MDSCs and activate the TGF-β signaling pathway to induce CAFs to secrete IL-6, jointly constructing an immune-privileged microenvironment [29,30]. This also explains why patients with high GDF6 expression have poor responses to anti-PD-1 therapy. On the other hand, GDF6 showed pro-immune activation, with its expression being positively correlated with PD-L1 immune treatment response. The possible mechanisms include GDF6 upregulating MHC-II expression through HIF-1α, enhancing antigen presentation [31,32], and that activating the VEGF-A pathway promotes blood vessel normalization and improves T-cell infiltration [33]. This “double-edged sword” characteristic suggests that targeted strategies need to be designed based on cancer-specific microenvironments.

Tumor genomic heterogeneity is a significant factor in therapeutic resistance. In this study, we found that GDF6 expression was significantly positively correlated with tumor mutational burden (TMB) in colorectal cancer (COAD), microsatellite instability (MSI) in testicular germ cell tumors (TGCT), and homologous recombination deficiency (HRD) in lung adenocarcinoma (LUAD). Mechanistically, GDF6 may exacerbate genomic instability through the following pathways: dependent on TGF-β pathway, thereby weakening DNA damage repair capabilities [34]; activating ROS production and inducing oxidative stress-related mutations [35]. Additionally, the upregulation of co-expression of m6A-modifying enzymes in GDF6-high-expressing tumors suggests that GDF6 may amplify oncogenic signals through epitranscriptomic regulation.

Enrichment analysis indicated that GDF6 co-expressed genes were significantly enriched in the PI3K-Akt, HIF-1, and VEGF pathways. GDF6 may synergistically promote tumor immune suppression through these pathways. For instance, it can upregulate PD-L1 expression in tumor cells by activating Akt phosphorylation [36]. Alternatively, under hypoxic conditions, HIF-1α can induce the expression of VEGF, PD-L1, and CXCL12 [37,38,39]. In vitro experiments further confirmed its functions; GDF6 drives a metastatic phenotype, possibly by inhibiting the RhoA/ROCK signaling pathway and blocking the assembly of actin stress fibers [38]. GDF6 may also reduce extracellular matrix degradation by downregulating MMP9 expression [39]. Notably, GDF6 was positively correlated with the stemness index in low-grade glioma (LGG), while it was negatively correlated in breast cancer (BRCA), suggesting that it may regulate tumor stem cell characteristics through tissue-specific pathways, such as the WNT or NOTCH pathways [40].

Although this study has revealed the multifaceted roles of GDF6, its clinical translation still faces challenges. In terms of prognostic stratification, patients with high GDF6 expression may benefit from anti-PD-L1 therapy but are resistant to anti-PD1 treatment. In combination therapy, for GDF6-high-expressing tumors, the combination of MDSC inhibitors (such as SX-682) [41] or PARP inhibitors (in HRD-positive cancer types) [42] may reverse immune suppression. However, this experiment has certain limitations. First, the reliance on public databases lacks single-cell resolution to parse the heterogeneity of the microenvironment. Second, in vitro models (such as htr8/svneo cells) do not fully simulate the biology of solid tumors. Third, the functional impact of GDF6 hotspot mutations has not been verified. Future research on GDF6 should focus on the following three aspects: (1) building conditional GDF6 knockout mouse models to parse its role in the formation of the pre-metastatic microenvironment; (2) screening small-molecule inhibitors or neutralizing antibodies targeting GDF6 to develop the clinical therapeutic applications of GDF6; (3) conducting prospective clinical trials to assess the feasibility of using GDF6 expression to guide immunotherapy decisions.

Through a comprehensive literature review, we identified several downstream effectors of GDF6, including VEGFA, FLT1 (VEGFR1), KDR (VEGFR2), Src, PD-L1, and CXCL12 [13,14,27,43,44]. Notably, Krispin et al. demonstrated that GDF6 influences vascular stability by restricting VEGF signaling, positioning VEGFA and KDR as critical downstream components of this pathway [13]. Additionally, Zhou et al. provided evidence that GDF6 modulates Src activity in Ewing sarcoma, further establishing Src as a downstream effector [27]. While direct associations between GDF6 and FLT1 were not found in the literature, Gao et al. utilized bioinformatics to predict that overexpressed miRNAs target both FLT1 and GDF6, suggesting a potential indirect link [44]. Given GDF6’s role in immune regulation, it is plausible to hypothesize that it may influence immune-related molecules such as PD-L1 and CXCL12 through TGF-β or BMP signaling pathways. Yang et al. elucidated the general mechanisms by which the TGF-β family contributes to immune evasion [14], and Ehata et al. reviewed the role of BMP signaling in cancer [43], indirectly supporting the notion that GDF6 may regulate tumor immune microenvironment modulators like PD-L1 and CXCL12. Future investigations into the relationship between GDF6 and these downstream effectors could enhance our understanding of the context-dependent mechanisms by which GDF6 regulates metastasis and immune evasion in malignancies.

As a member of the TGF-β superfamily, GDF6 exerts pleiotropic effects on tumor progression by regulating the immune microenvironment, genomic stability, and multiple signaling pathway networks. Its expression heterogeneity, dual-edged sword characteristic in treatment response, and mechanistic complexity call for future studies to design precision intervention strategies in the context of cancer-specific backgrounds. By integrating molecular biology, immunology, and clinical medicine across disciplines, GDF6 holds promise as a new target for tumor individualized treatment, propelling the development of precision oncology.

## 5. Conclusions

This pan-cancer analysis reveals GDF6 as a dual-function regulator with tissue-specific roles in tumorigenesis. GDF6 exhibits bidirectional dysregulation: downregulation in 23 cancers (e.g., GBM, BRCA) and upregulation in 7 subtypes (e.g., KIRC, PAAD), correlating with advanced stages (KIRP, STAD) and poor survival (OS/DFS in KIRP, STAD, MESO). Mechanistically, GDF6 drives metastasis via PI3K-Akt/HIF-1/VEGF pathways, recruits immunosuppressive cells (MDSCs, CAFs), and amplifies genomic instability. Paradoxically, GDF6-high tumors resist anti-PD1 but respond better to anti-PD-L1, highlighting its potential as an immunotherapy biomarker. Functional validation confirmed GDF6’s pro-migratory role in vitro. Despite limitations in bulk omics data, this study positions GDF6 as a pivotal orchestrator of tumor progression, immune modulation, and therapy resistance. Future work should prioritize GDF6-targeted therapies (e.g., inhibitors/antibodies) and clinical trials to translate its prognostic/predictive potential into precision oncology strategies.

## Data Availability

Data used to verify the validity of biomarkers in the study originated from the GEO, with the specific serial numbers shown in the figure legend.

## Supplementary Materials

The following supporting information can be downloaded at: https://www.mdpi.com/article/10.3390/cimb47040249/s1, Table S1: 100 most-similar genes of GDF6; Figure S1: GDF6 expression across molecular subtypes in breast cancer (BRCA) (A), colorectal carcinoma and rectal adenocarcinoma (COADREAD) (B), glioblastoma (GBM) (C) and uterine corpus endometrial carcinoma (UCEC) (D); Figure S2: Distribution of GDF6 protein expression levels across various cancer types based on immunohistochemistry (IHC) staining data retrieved from the Human Protein Atlas (HPA) database; Figure S3: Correlation between GDF6 expression and the pathological stages of BLCA, HNSC, KIRC, UCS, LIHC, SCKM, UCEC, STAD, and KIRP; Figure S4: Forest plots of hazard ratios for GDF6 expression and overall survival (OS) (A), disease-specific survival (DSS) (B), disease-free interval (DFI) (C), and progression-free interval (PFI) (D) in multiple cancers; Figure S5: Comparison of GDF6 gene expression levels between different tumor models and ICB treatment, before and after ICB treatment, and between responders and non-responders; File S1: Microscopy images and migration rate.
