# Supplementary material for: Dual-Faced Role of GDF6 in Cancer: Mechanistic Insights into Its Context-Dependent Regulation of Metastasis and Immune Evasion Across Human Malignancies
Source: Curr Issues Mol Biol. 2025 Apr 2;47(4):249. doi: 10.3390/cimb47040249 (PMC12025365; doi:10.3390/cimb47040249)
Supplement: Supplementary file 1 [file cimb-47-00249-s001.zip › Supplementary Materials Figures S1-S5.pdf]

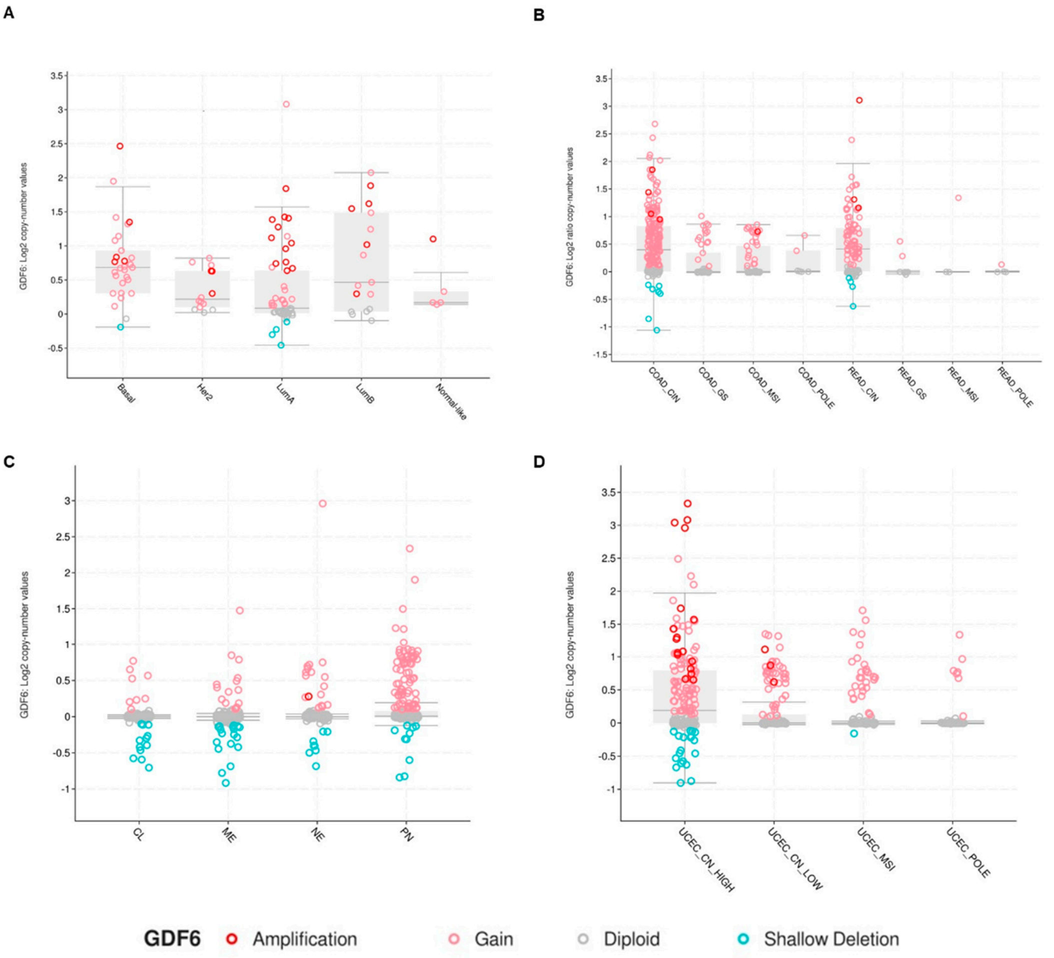

Figure S1. GDF6 expression across molecular subtypes in breast cancer (BRCA) (**A**), colorectal carcinoma and rectal adenocarcinoma (COADREAD) (**B**), glioblastoma (GBM) (**C**) and uterine corpus endometrial carcinoma (UCEC) (**D**).

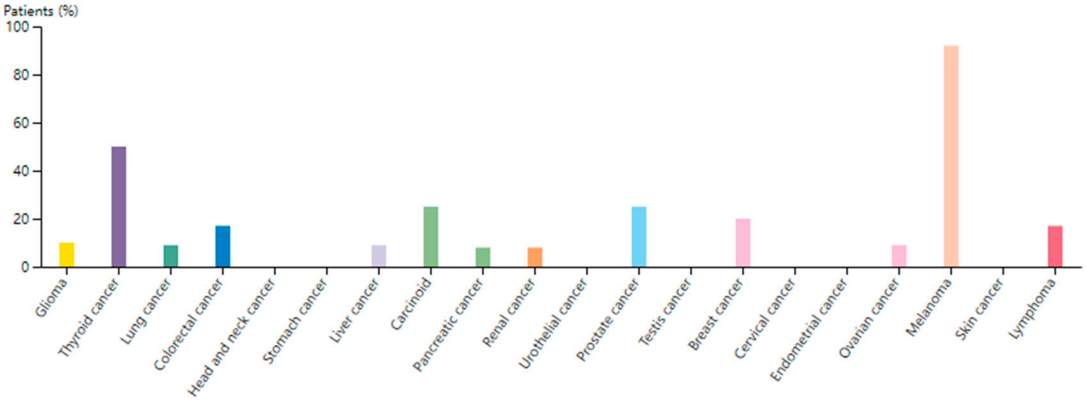

Figure S2. Distribution of GDF6 protein expression levels across various cancer types based on immunohistochemistry (IHC) staining data retrieved from the Human Protein Atlas (HPA) database.

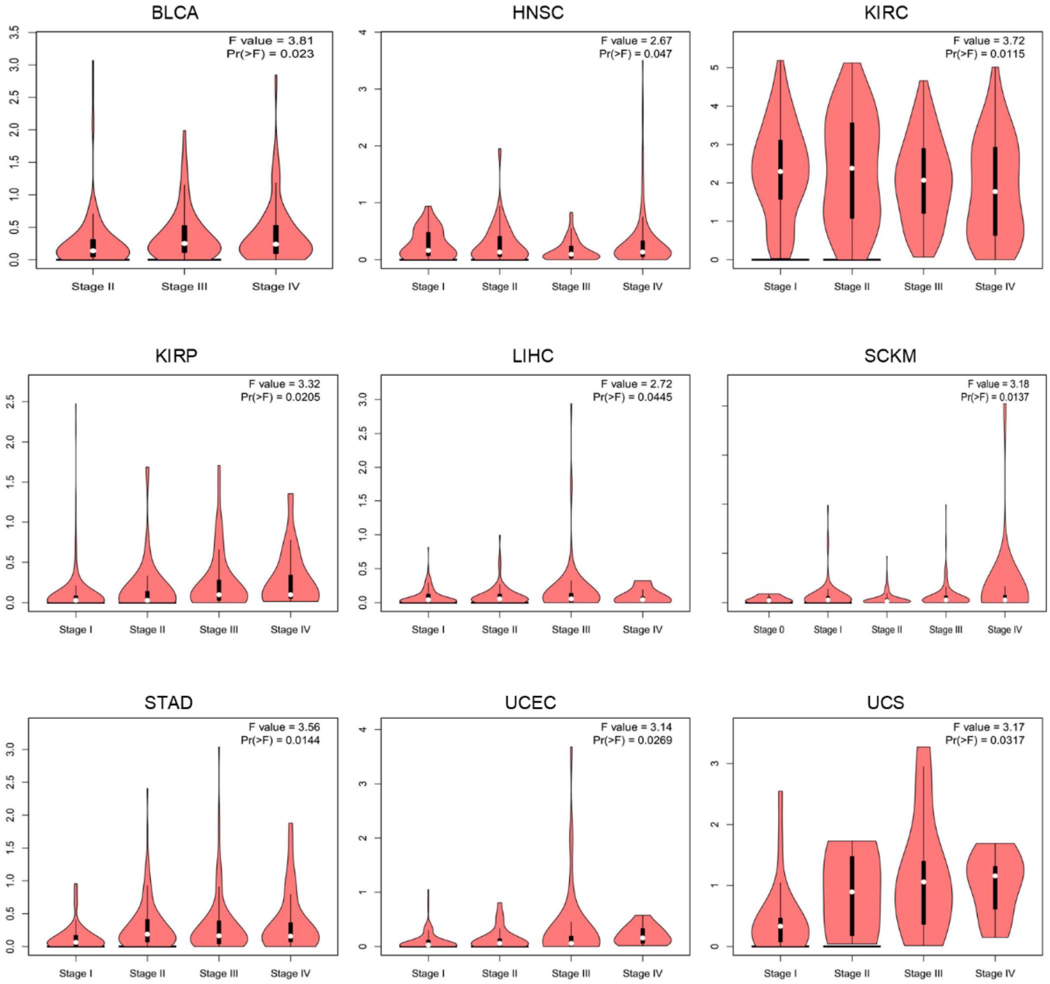

Figure S3. Correlation between GDF6 expression and the pathological stages of BLCA, HNSC, KIRC, UCS, LIHC, SCKM, UCEC, STAD, and KIRP.

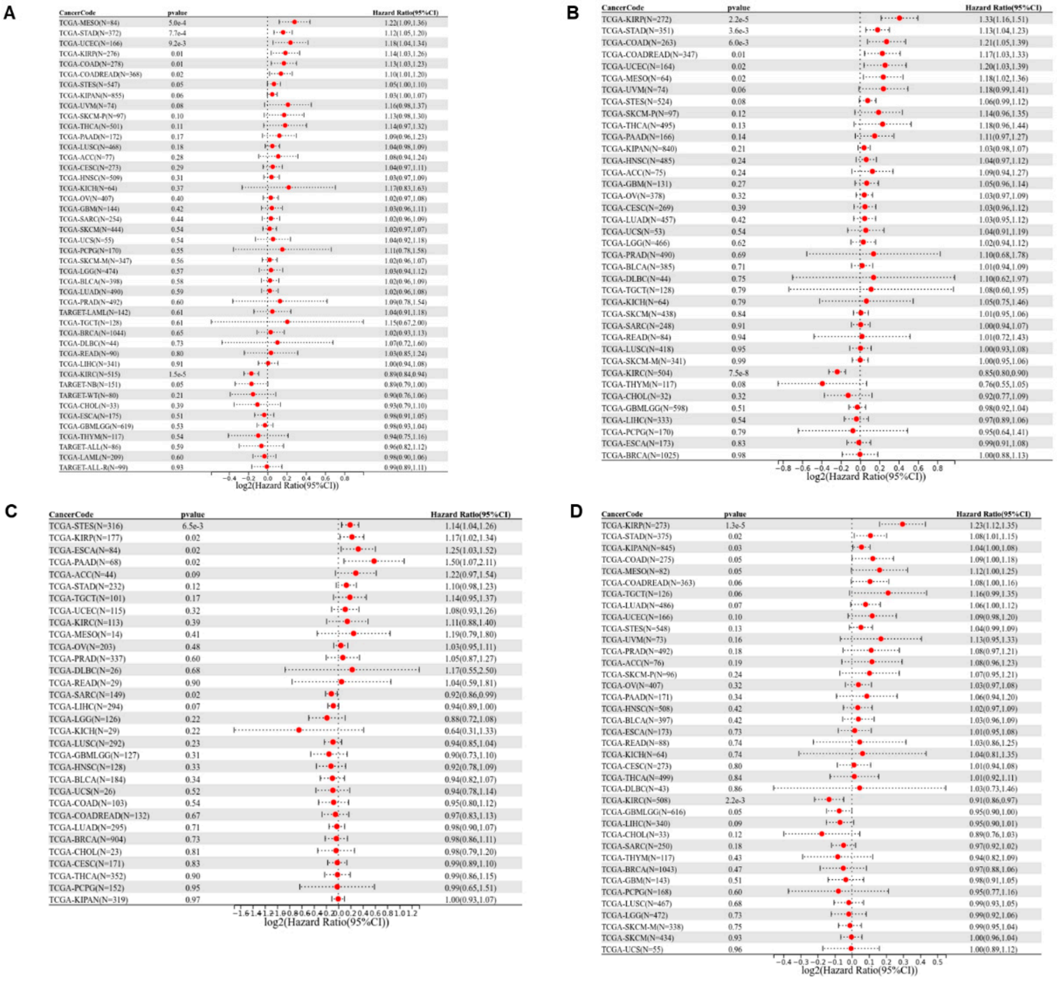

Figure S4. Forest plots of hazard ratios for GDF6 expression and overall survival (OS) (**A**), disease-specific survival (DSS) (**B**), disease-free interval (DFI) (**C**), and progression-free interval (PFI) (**D**) in multiple cancers.

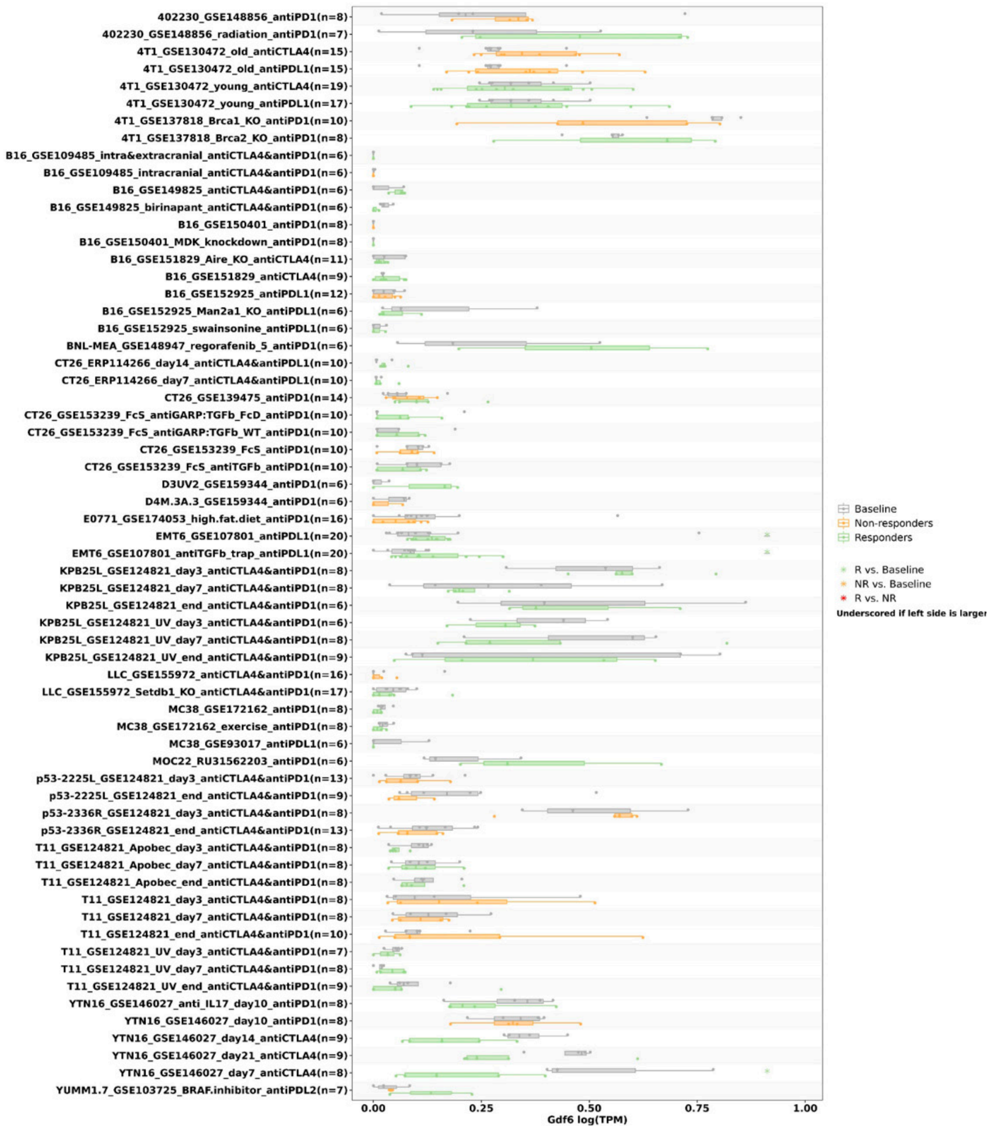

Figure S5. Comparison of GDF6 gene expression levels between different tumor models and ICB treatment, before and after ICB treatment, and between responders and non-responders. * $p < 0.05$.
