# Supplementary figures and images for: Dual-Faced Role of GDF6 in Cancer: Mechanistic Insights into Its Context-Dependent Regulation of Metastasis and Immune Evasion Across Human Malignancies
Source: Curr Issues Mol Biol. 2025 Apr 2;47(4):249. doi: 10.3390/cimb47040249 (PMC12025365; doi:10.3390/cimb47040249)

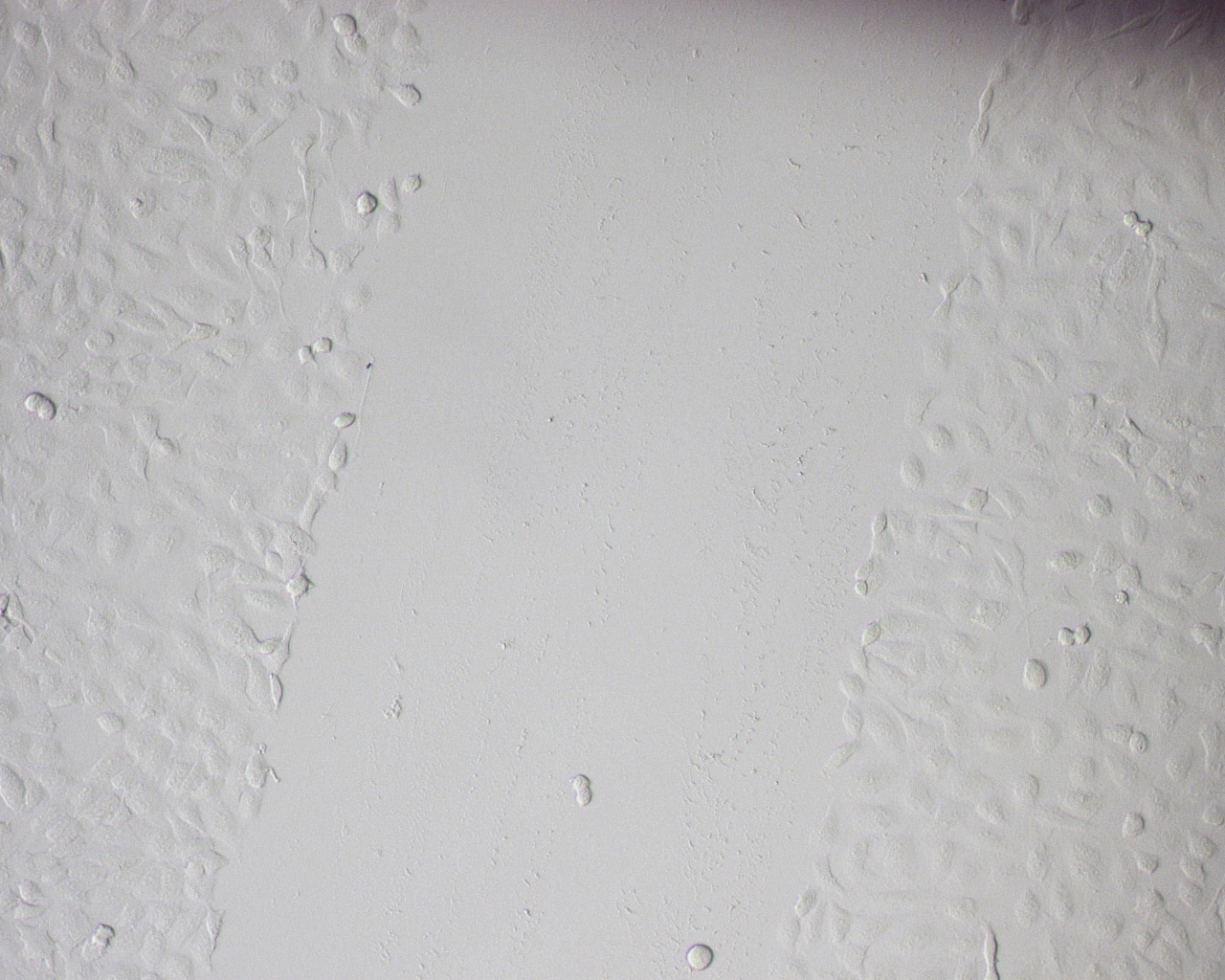

Supplement: Supplementary file 1 [file cimb-47-00249-s001.zip › File S1. Microscopy images and migration rate/0h-si-GDF6_1.jpg]

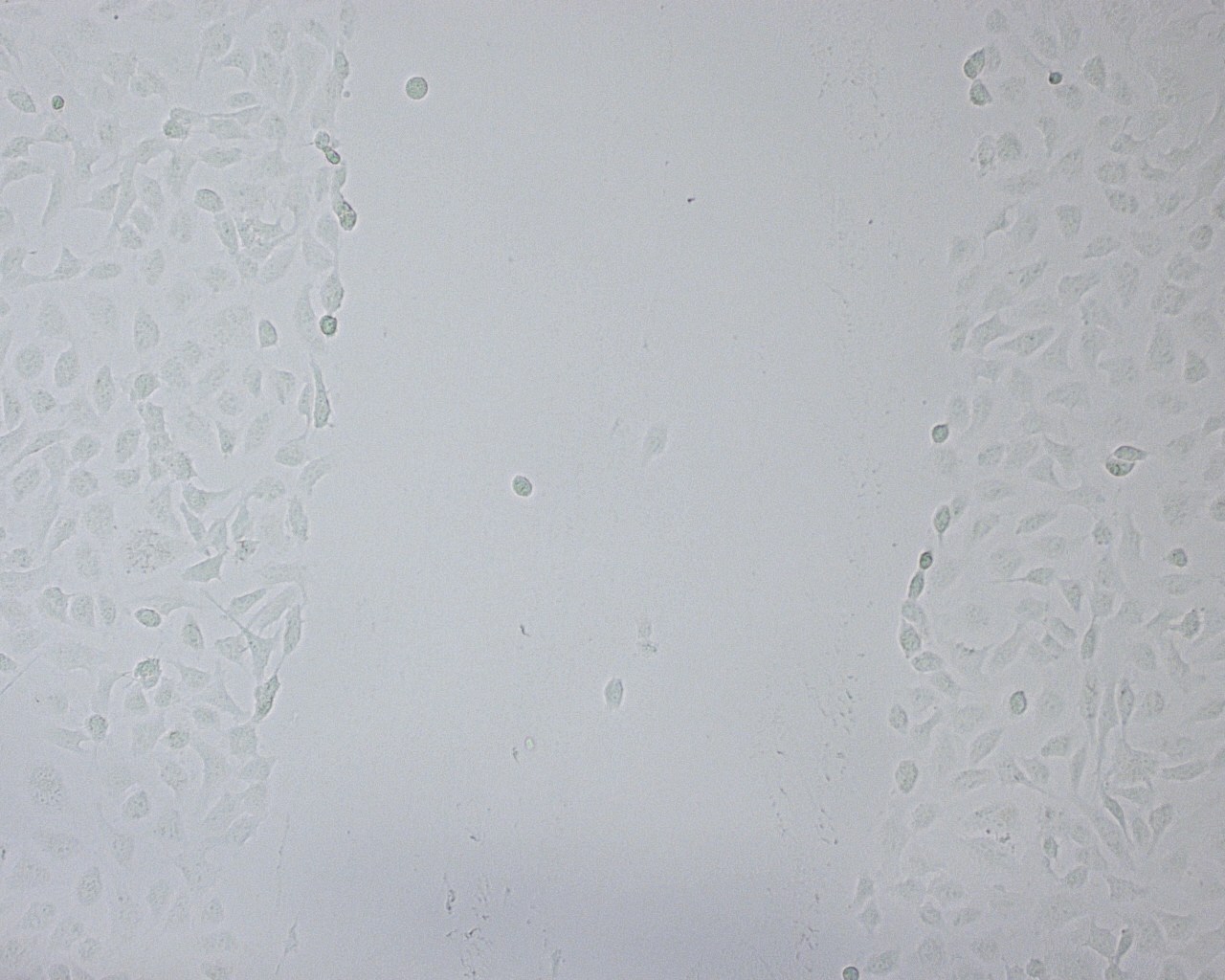

Supplement: Supplementary file 1 [file cimb-47-00249-s001.zip › File S1. Microscopy images and migration rate/0h-si-GDF6_2.jpg]

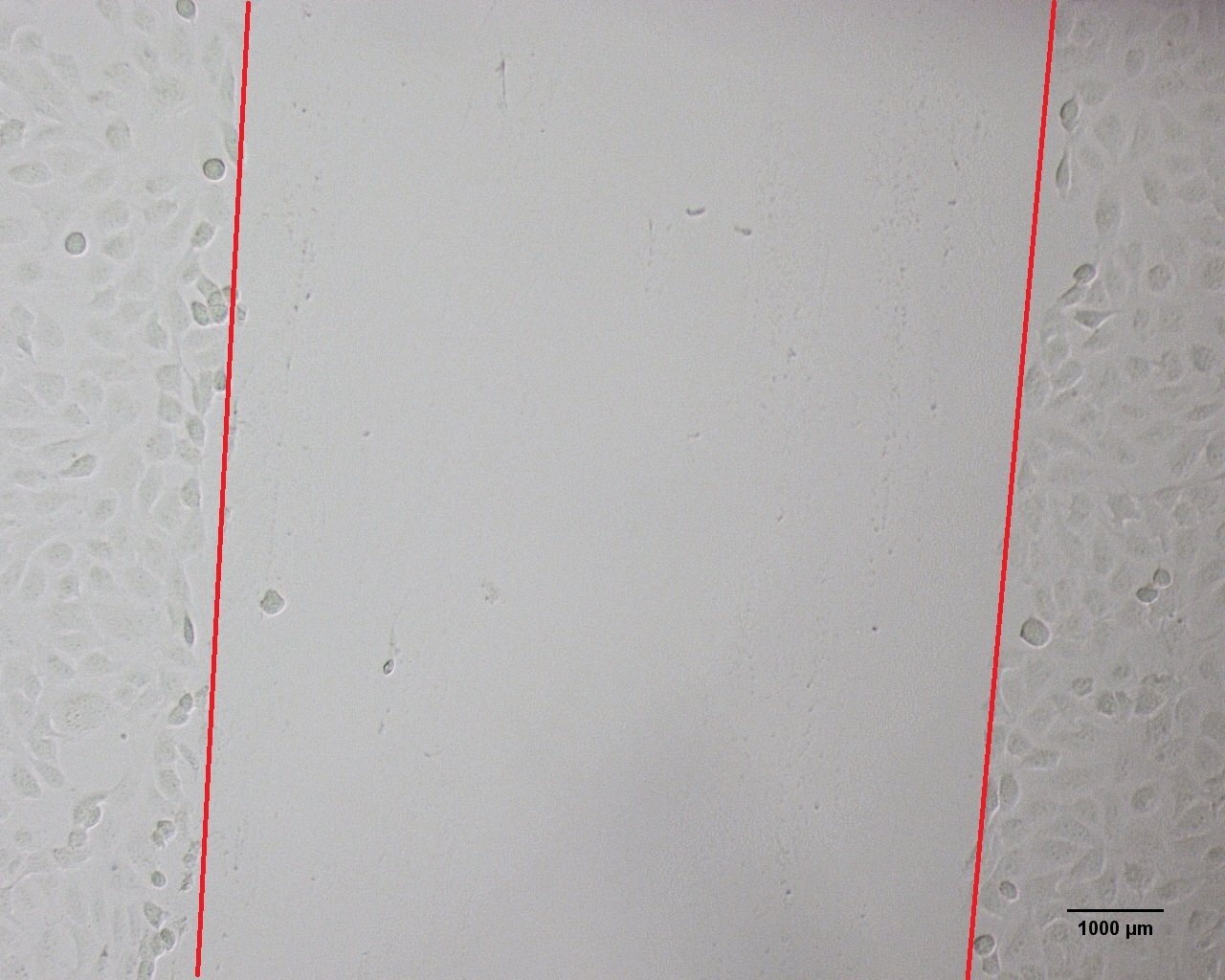

Supplement: Supplementary file 1 [file cimb-47-00249-s001.zip › File S1. Microscopy images and migration rate/0h-si-GDF6_3.jpg]

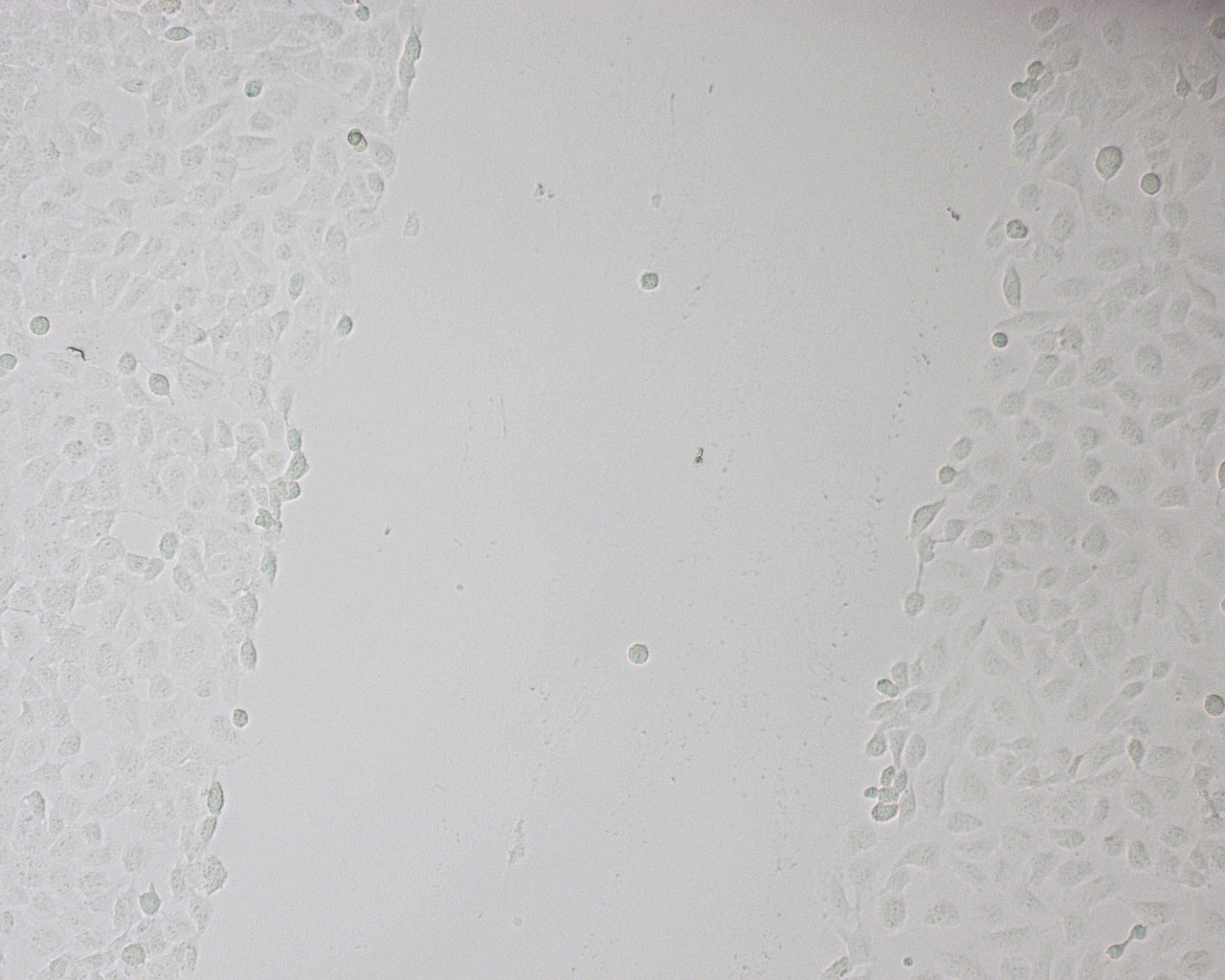

Supplement: Supplementary file 1 [file cimb-47-00249-s001.zip › File S1. Microscopy images and migration rate/0h-si-GDF6_4.jpg]

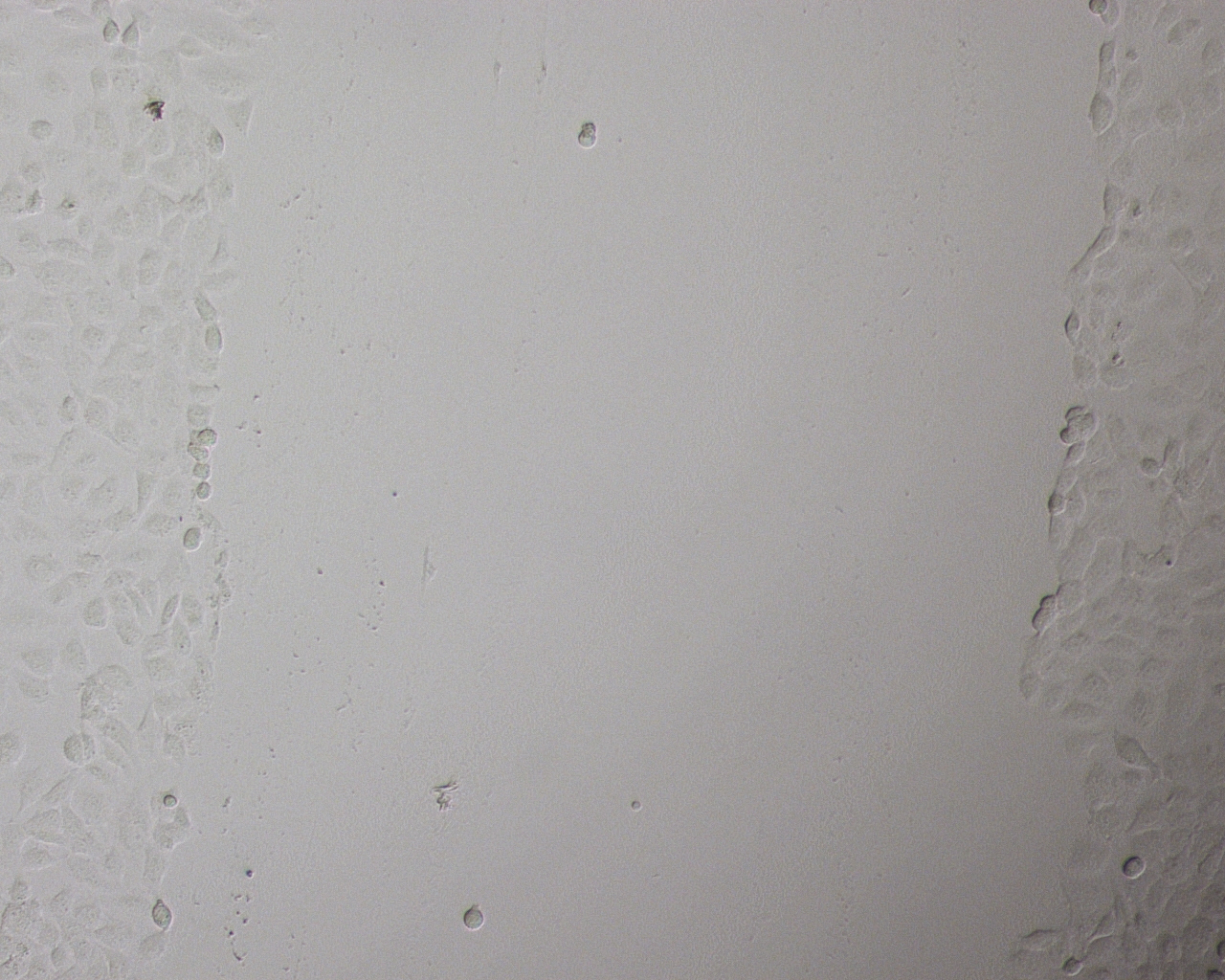

Supplement: Supplementary file 1 [file cimb-47-00249-s001.zip › File S1. Microscopy images and migration rate/0h-si-GDF6_5-Exclusion due to technical artifacts.jpg]

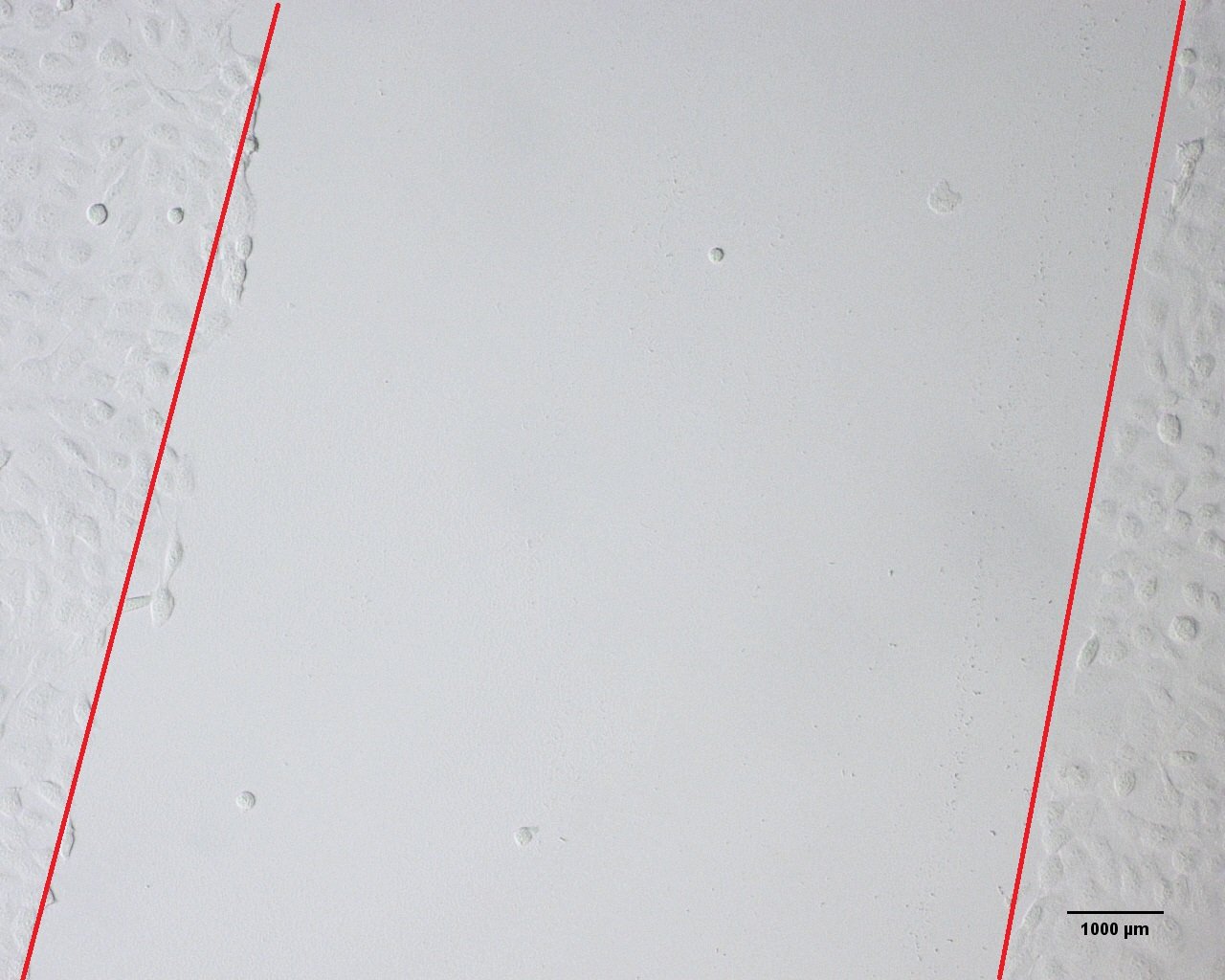

Supplement: Supplementary file 1 [file cimb-47-00249-s001.zip › File S1. Microscopy images and migration rate/0h-si-NC_1.jpg]

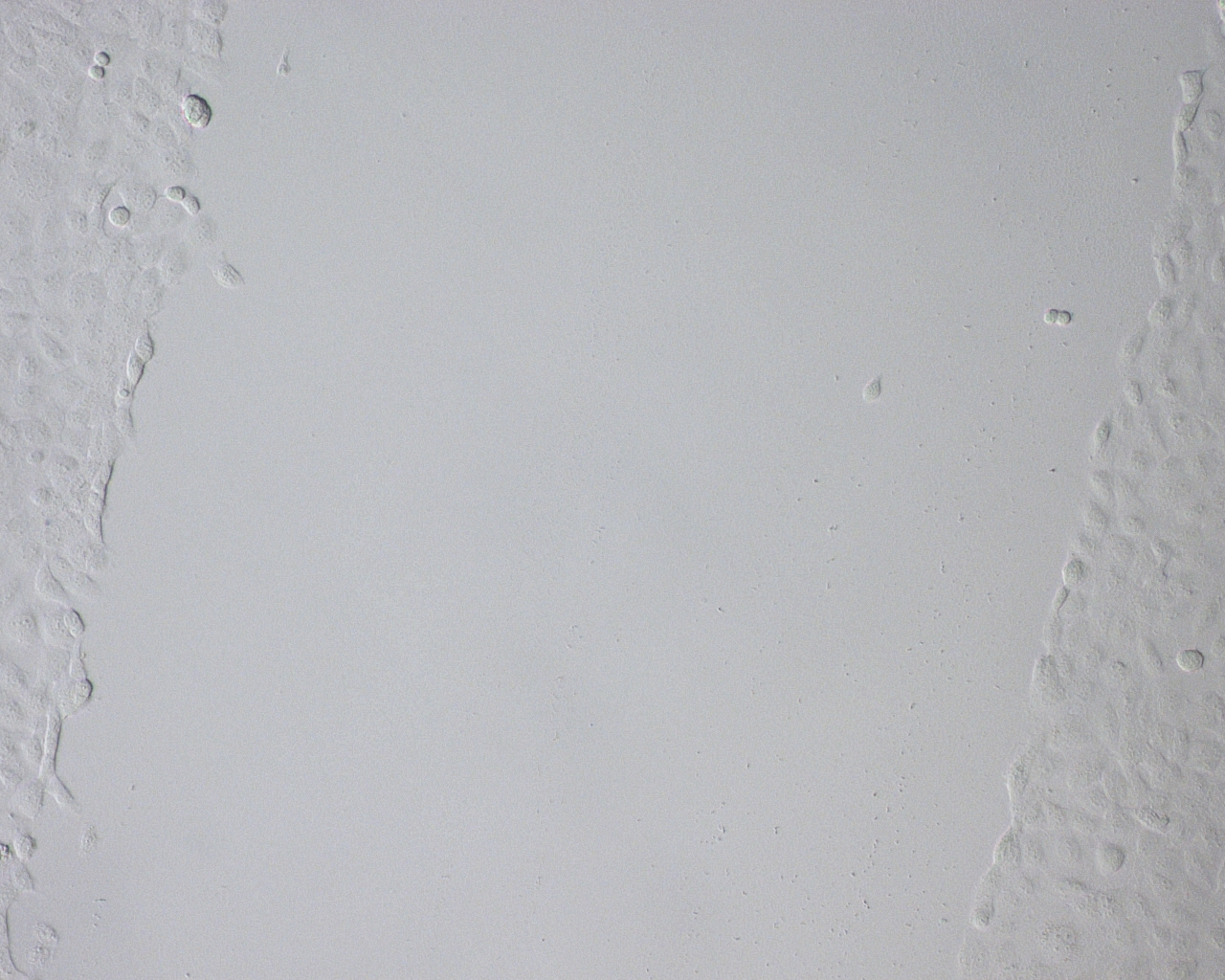

Supplement: Supplementary file 1 [file cimb-47-00249-s001.zip › File S1. Microscopy images and migration rate/0h-si-NC_2.jpg]

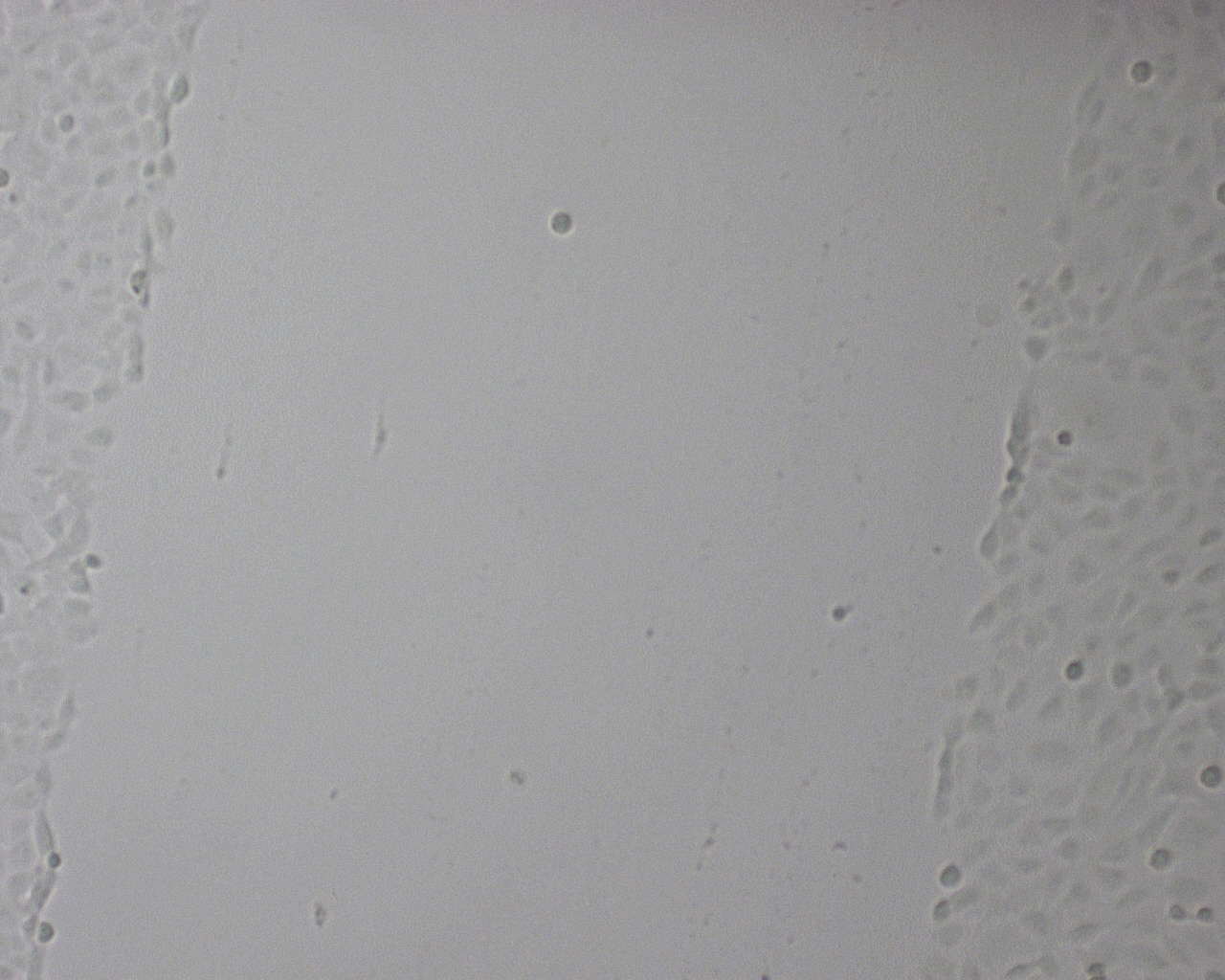

Supplement: Supplementary file 1 [file cimb-47-00249-s001.zip › File S1. Microscopy images and migration rate/0h-si-NC_3.jpg]

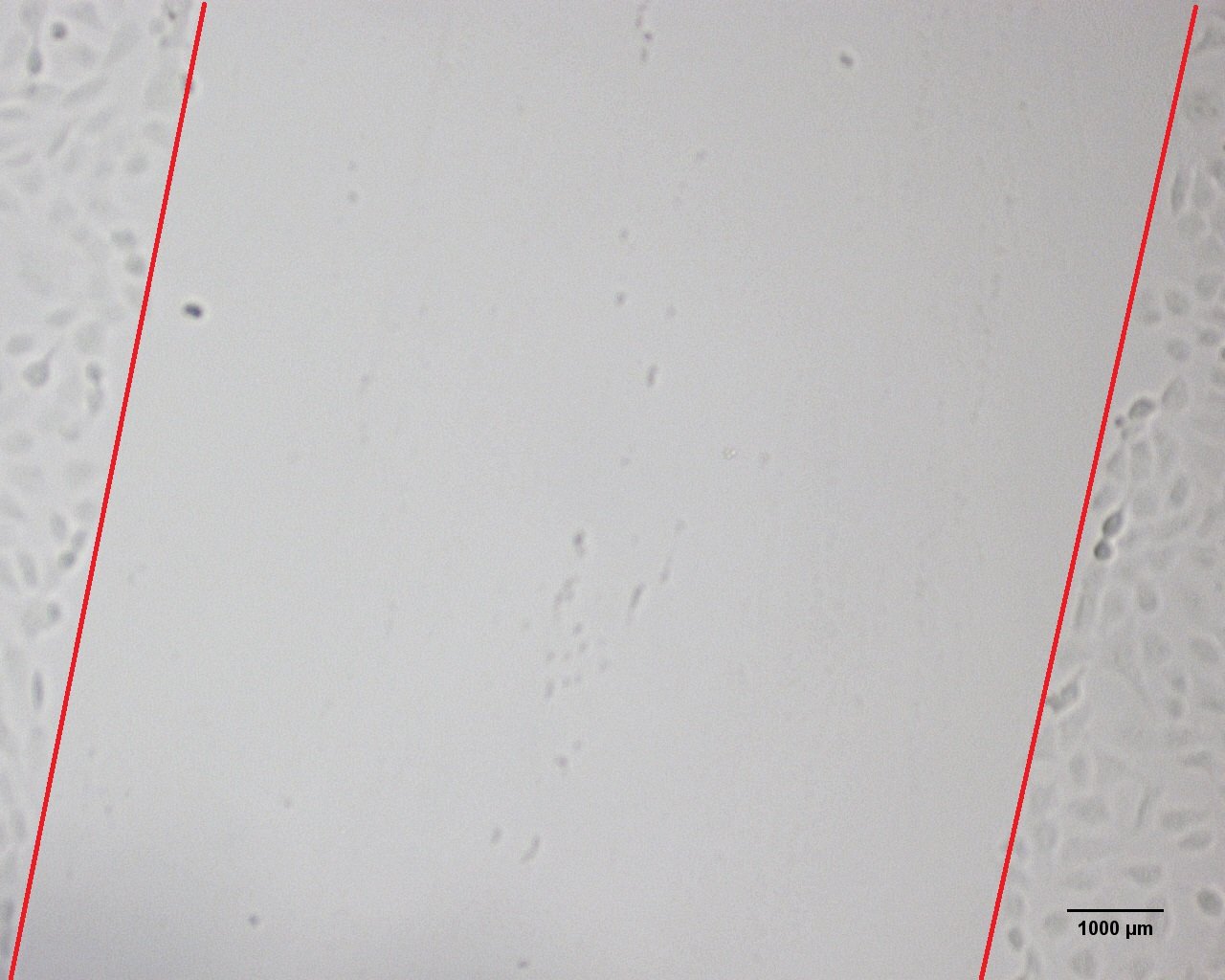

Supplement: Supplementary file 1 [file cimb-47-00249-s001.zip › File S1. Microscopy images and migration rate/0h-si-NC_4.jpg]

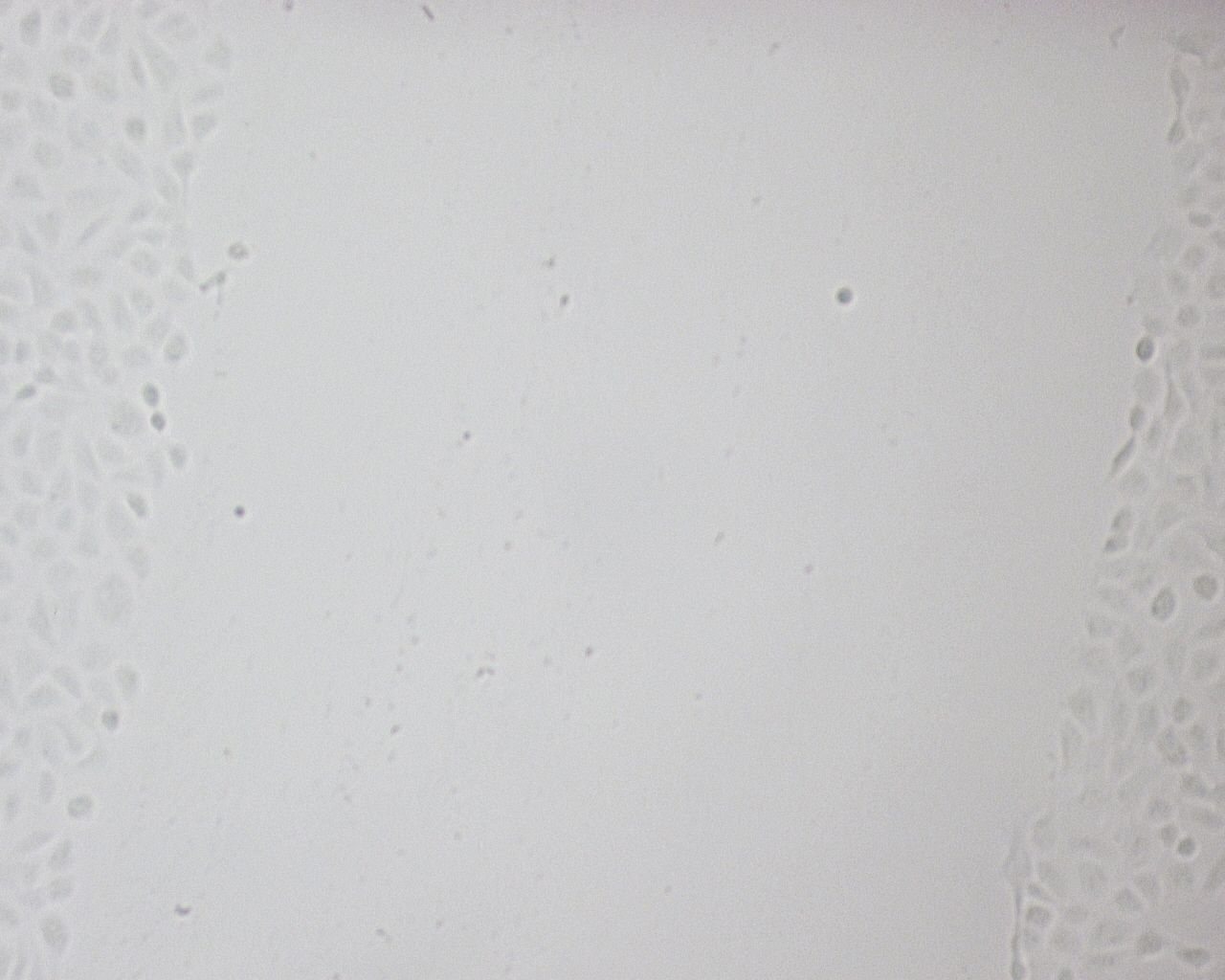

Supplement: Supplementary file 1 [file cimb-47-00249-s001.zip › File S1. Microscopy images and migration rate/0h-si-NC_5.jpg]

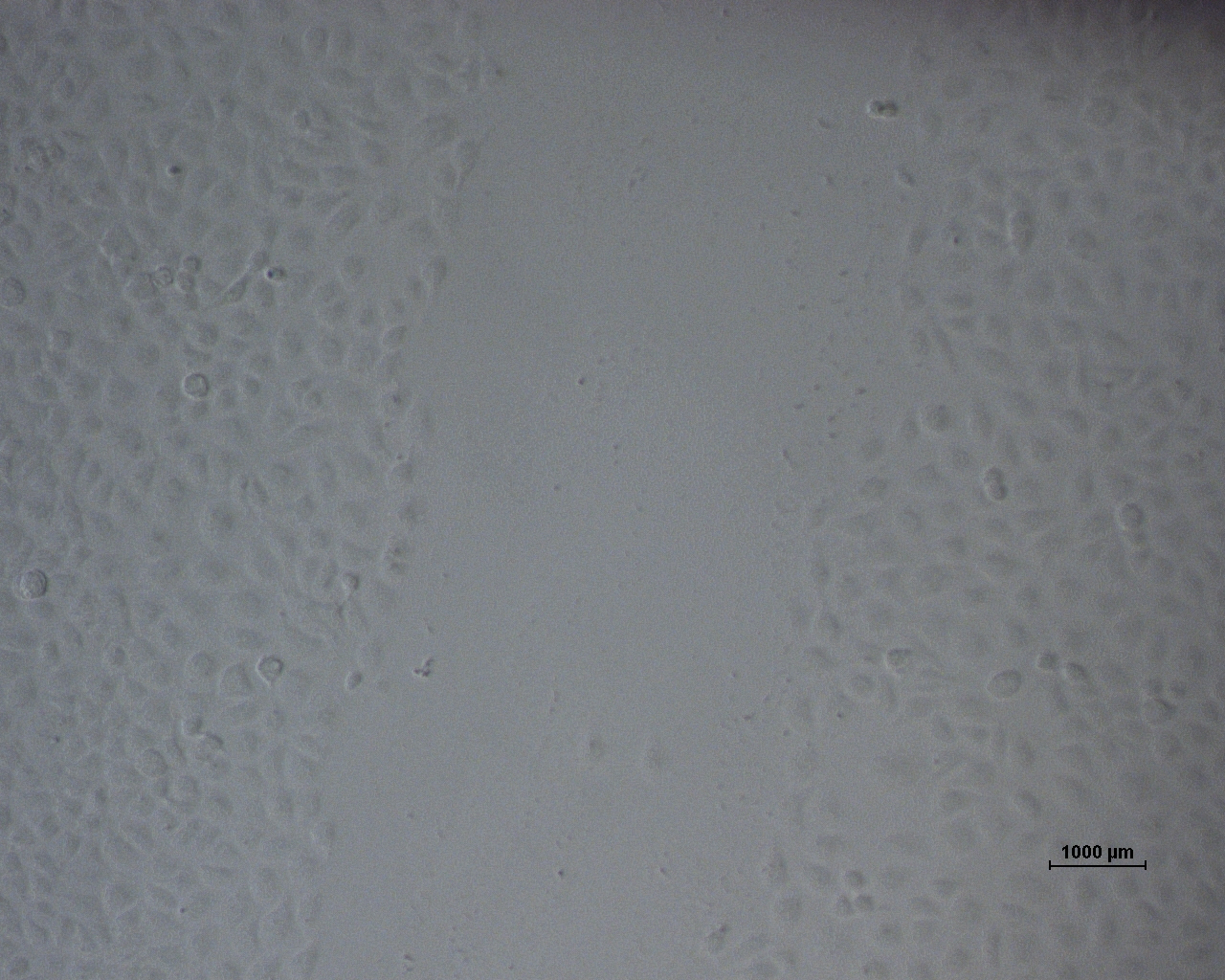

Supplement: Supplementary file 1 [file cimb-47-00249-s001.zip › File S1. Microscopy images and migration rate/6h-si-GDF6_1.jpg]

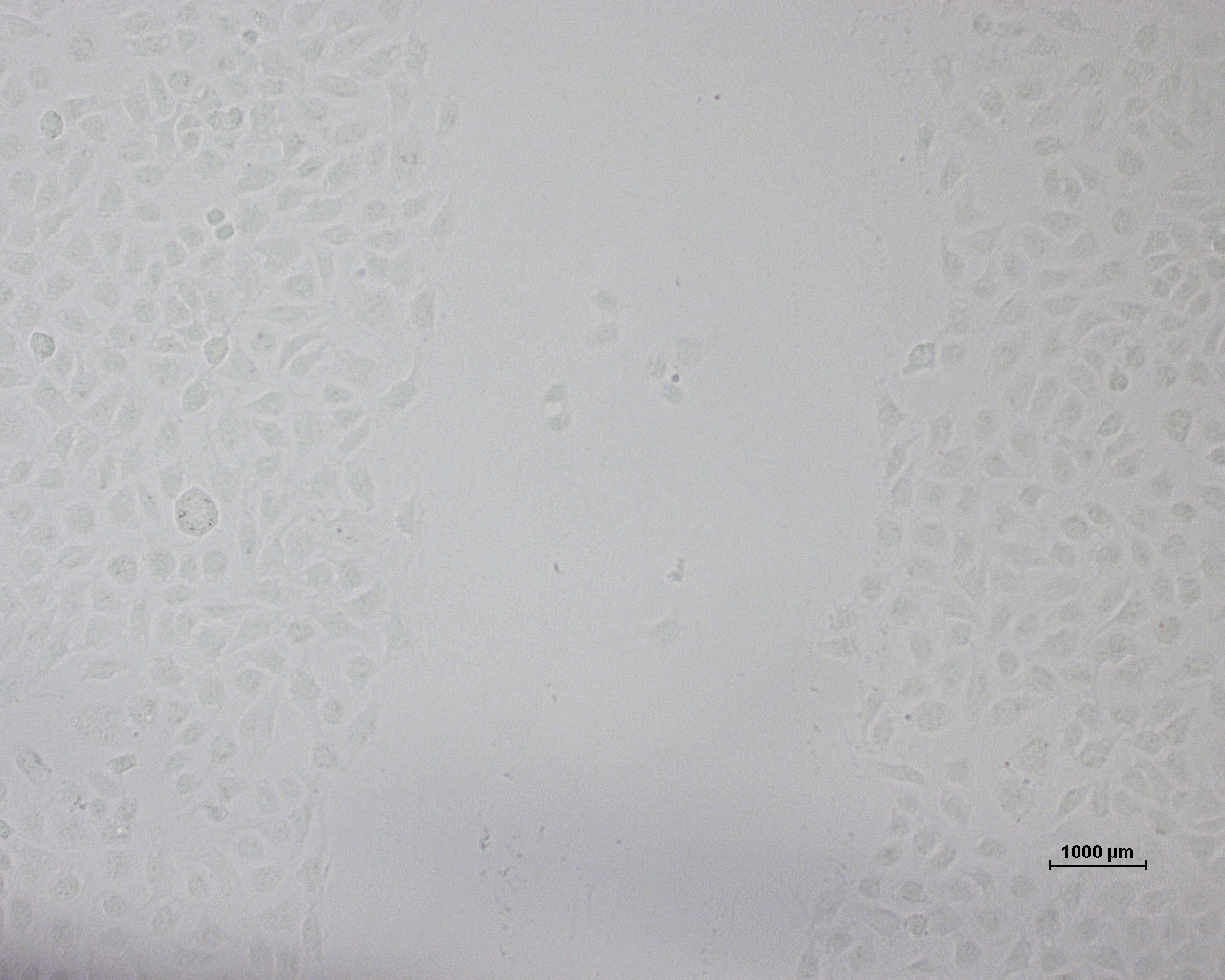

Supplement: Supplementary file 1 [file cimb-47-00249-s001.zip › File S1. Microscopy images and migration rate/6h-si-GDF6_2.jpg]

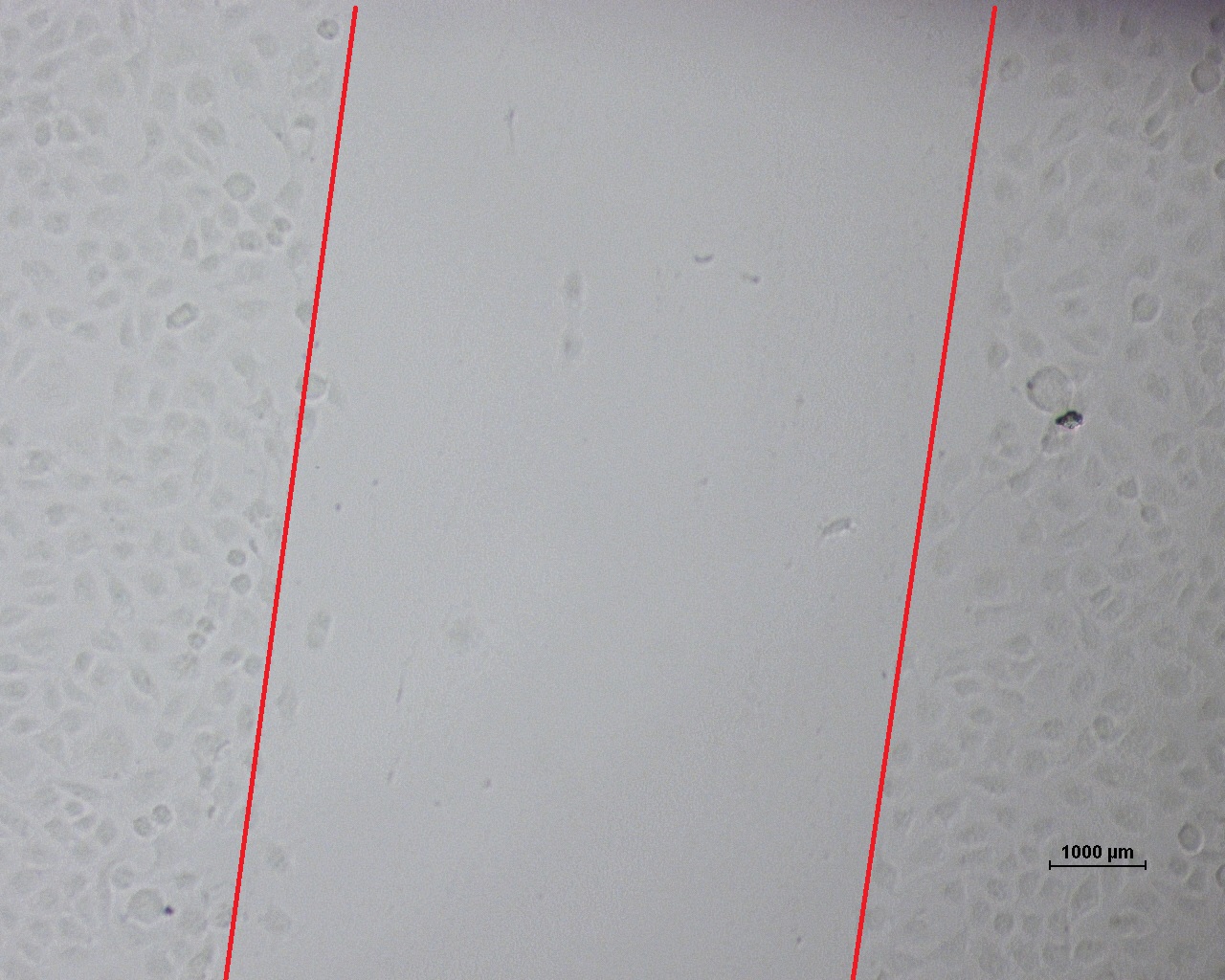

Supplement: Supplementary file 1 [file cimb-47-00249-s001.zip › File S1. Microscopy images and migration rate/6h-si-GDF6_3.jpg]

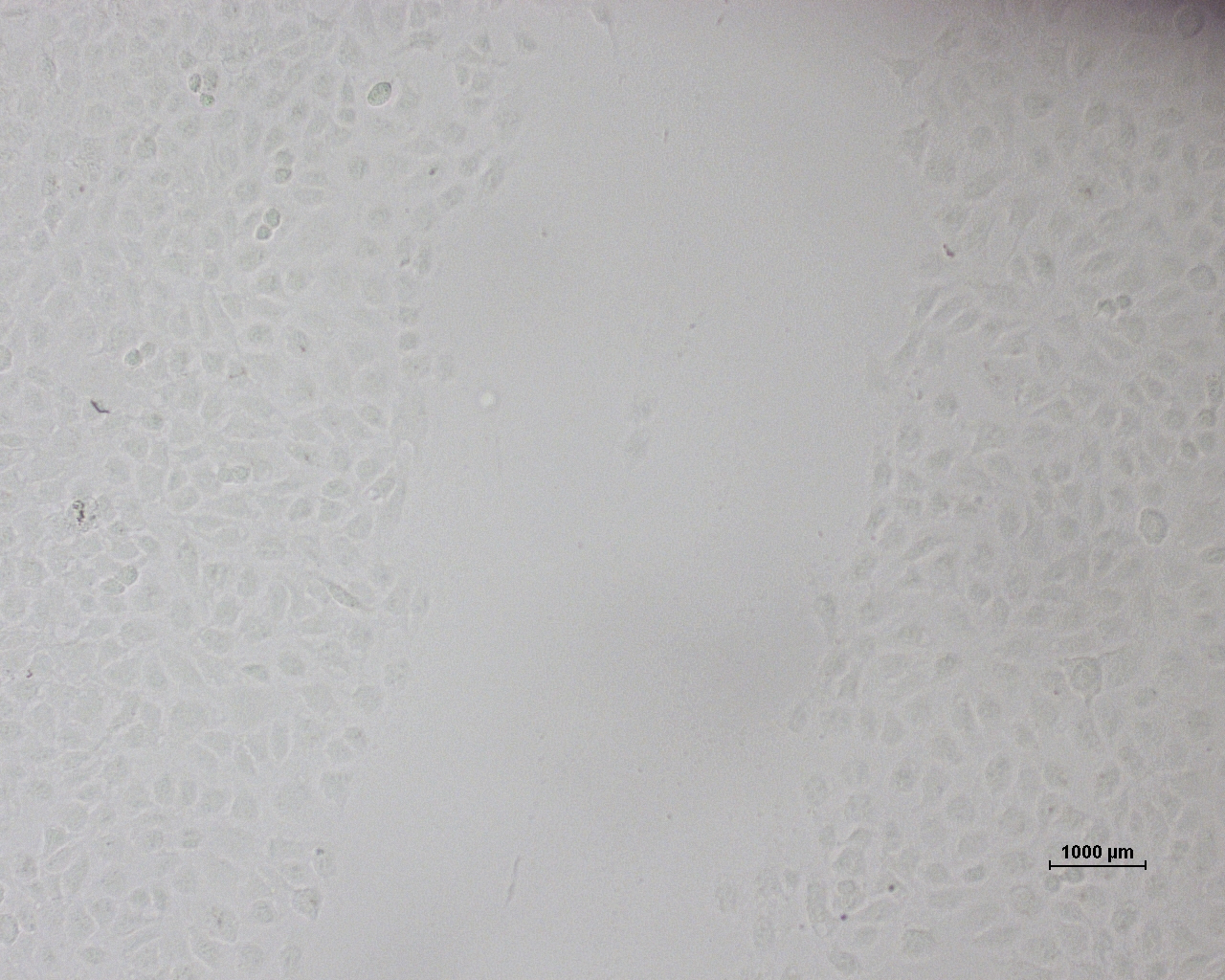

Supplement: Supplementary file 1 [file cimb-47-00249-s001.zip › File S1. Microscopy images and migration rate/6h-si-GDF6_4.jpg]

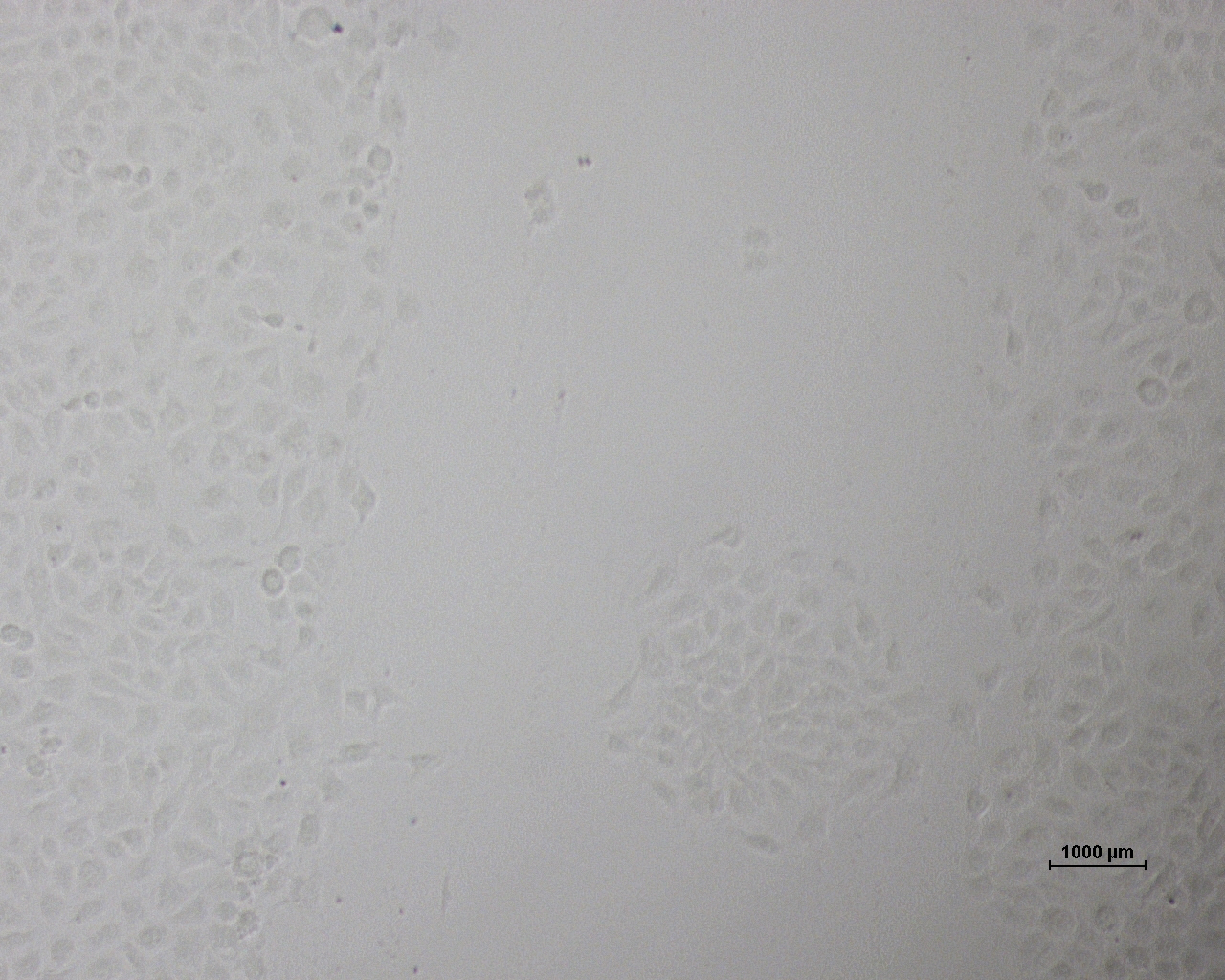

Supplement: Supplementary file 1 [file cimb-47-00249-s001.zip › File S1. Microscopy images and migration rate/6h-si-GDF6_5-Exclusion due to technical artifacts.jpg]

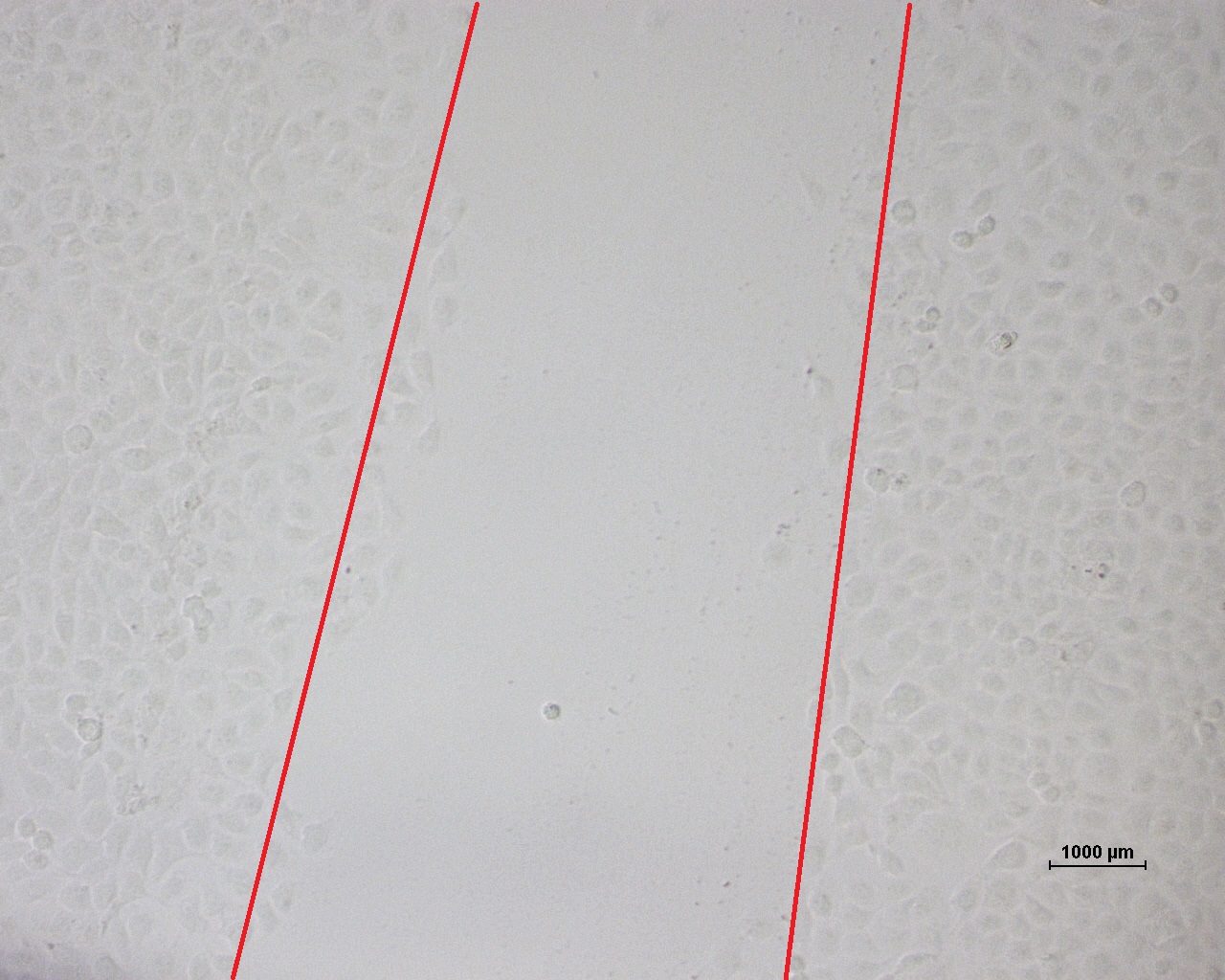

Supplement: Supplementary file 1 [file cimb-47-00249-s001.zip › File S1. Microscopy images and migration rate/6h-si-NC_1.jpg]

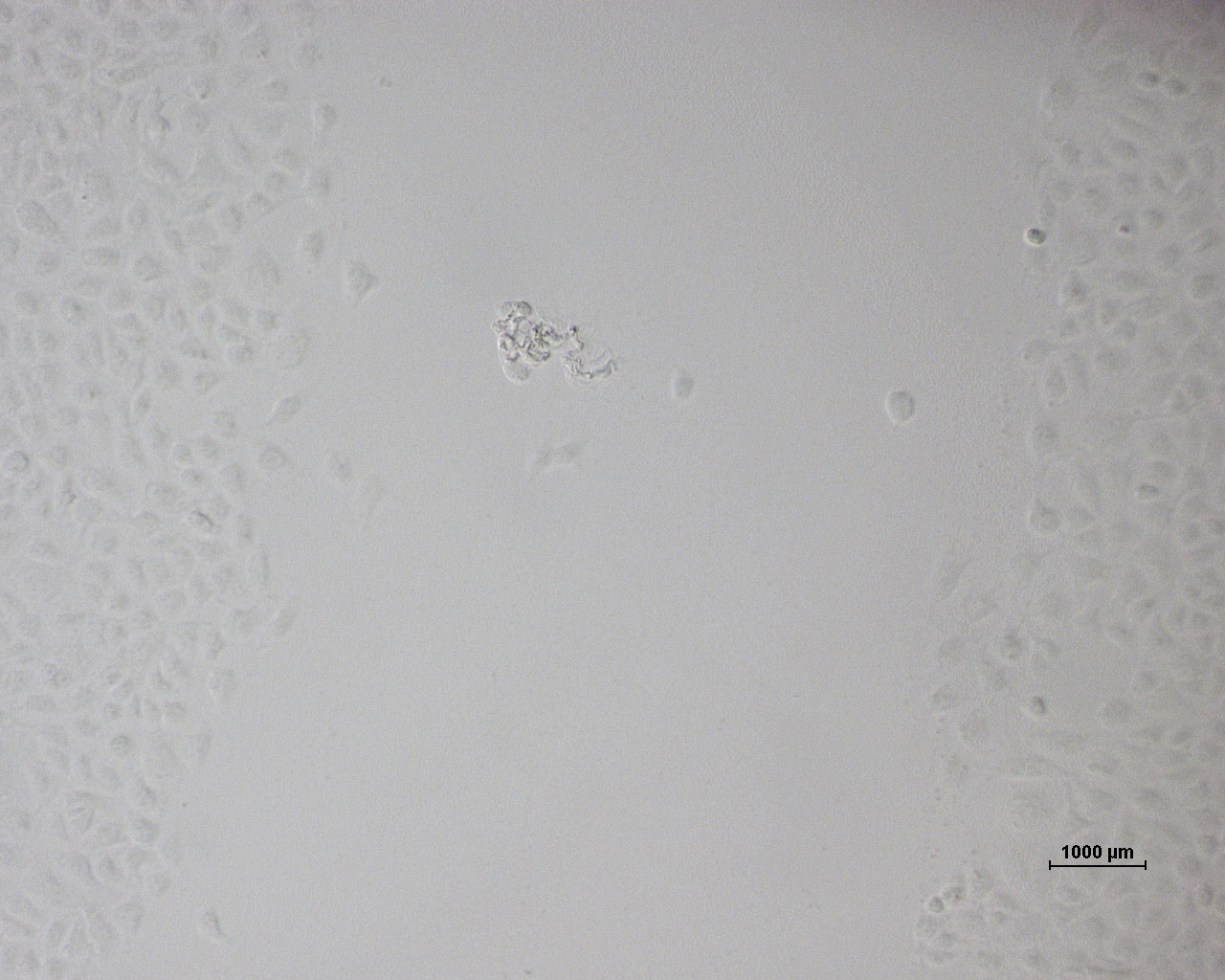

Supplement: Supplementary file 1 [file cimb-47-00249-s001.zip › File S1. Microscopy images and migration rate/6h-si-NC_2.jpg]

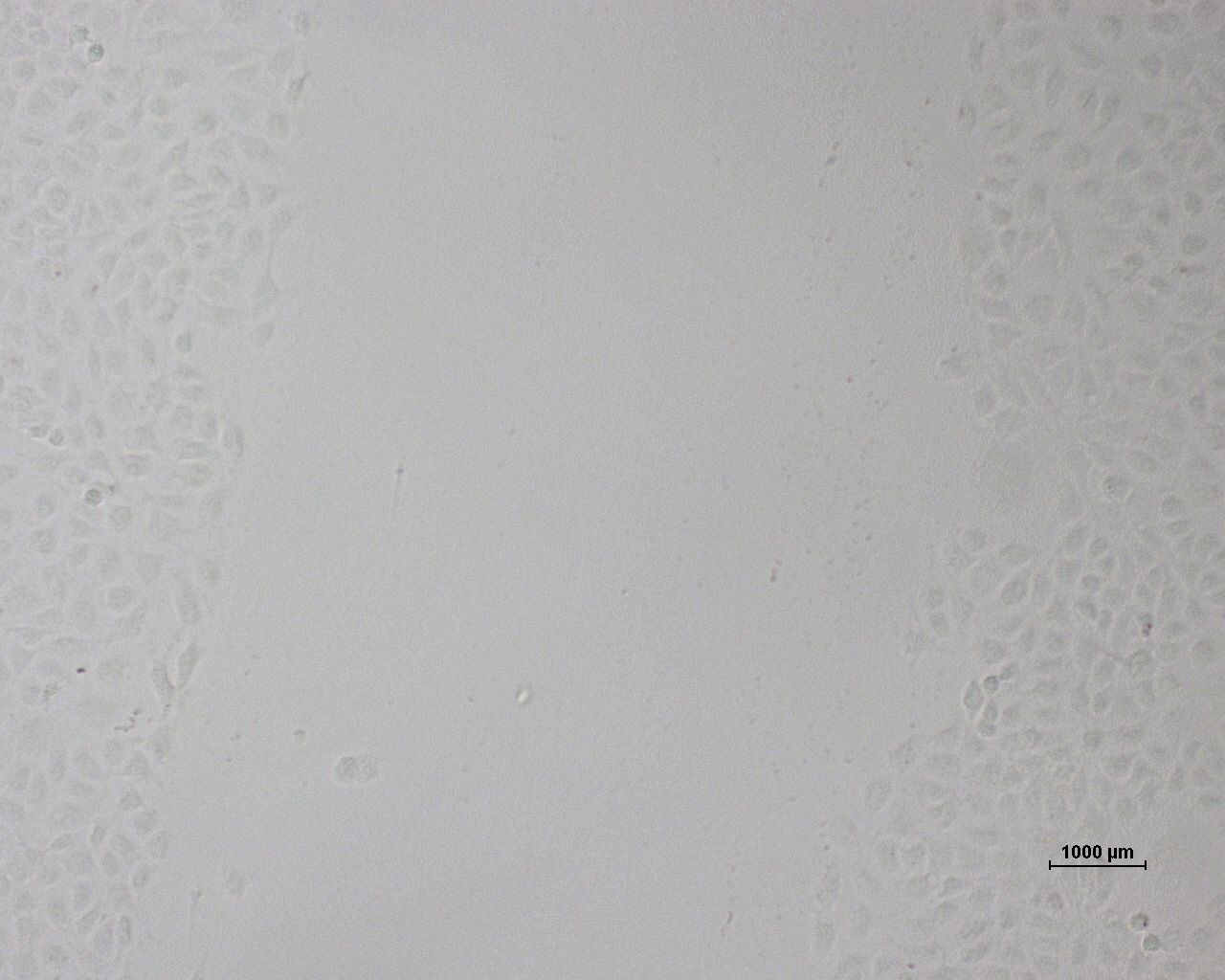

Supplement: Supplementary file 1 [file cimb-47-00249-s001.zip › File S1. Microscopy images and migration rate/6h-si-NC_3.jpg]

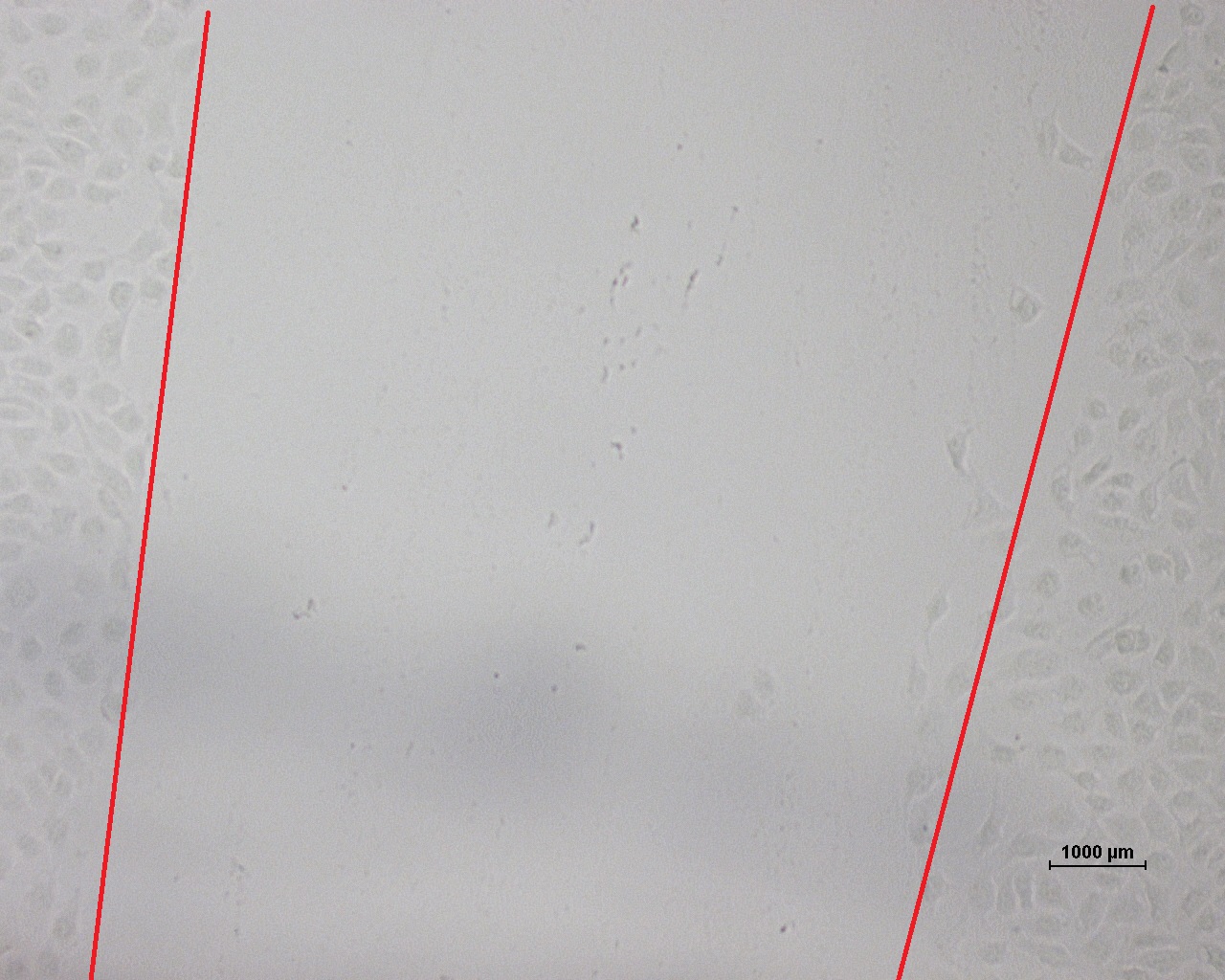

Supplement: Supplementary file 1 [file cimb-47-00249-s001.zip › File S1. Microscopy images and migration rate/6h-si-NC_4.jpg]

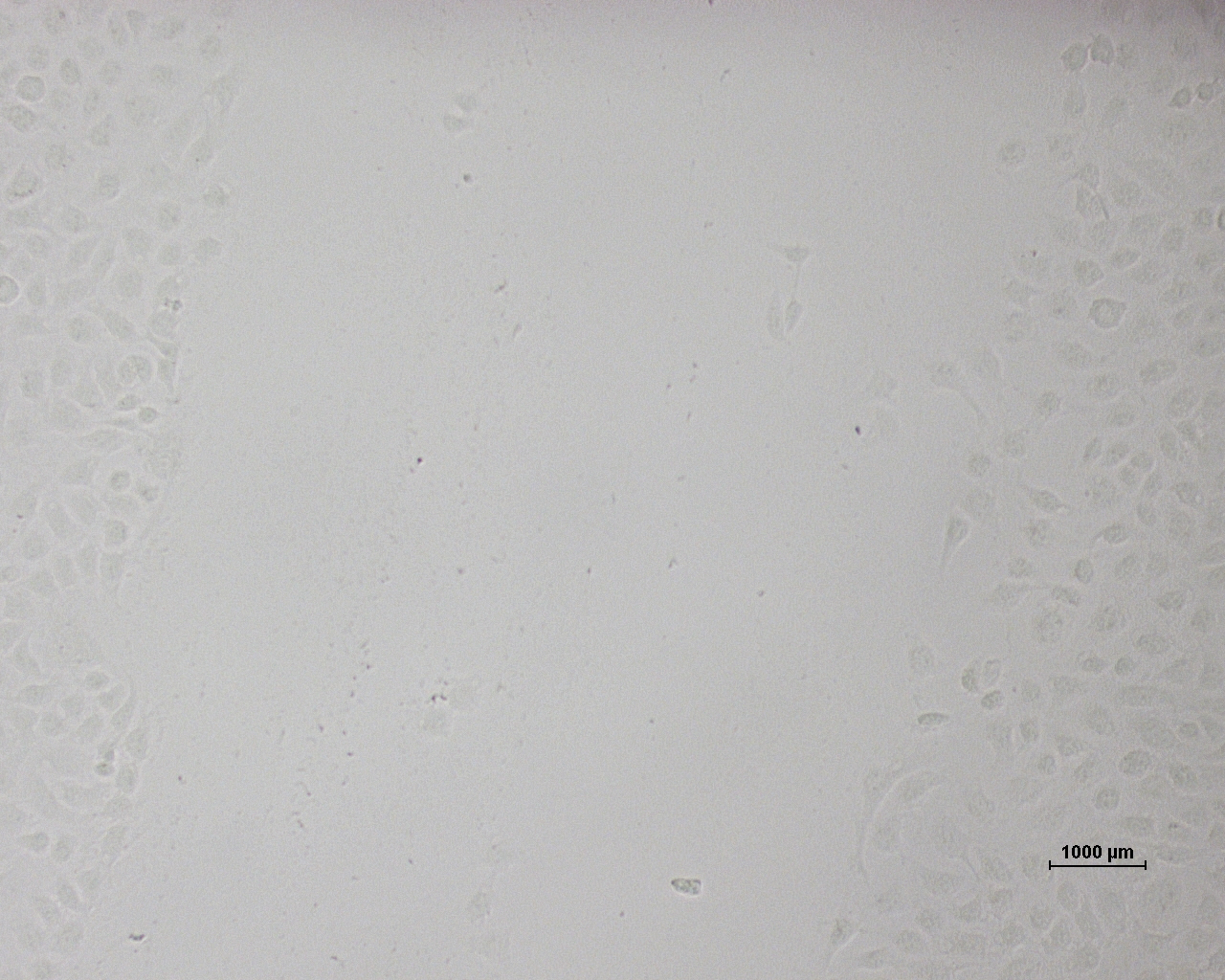

Supplement: Supplementary file 1 [file cimb-47-00249-s001.zip › File S1. Microscopy images and migration rate/6h-si-NC_5.jpg]
